# Within-sibling attenuation of polygenic risk score accuracy: investigating the effects of principal component analysis, LD score regression, and mixed model association in the UK Biobank

**DOI:** 10.1007/s00439-026-02852-3

**Published:** 2026-07-04

**Authors:** Ciaran Michael Kelly, Onyedika Onuorah, Edmund Gilbert

**Affiliations:** 1 School of Pharmacy and Biomolecular Sciences, RCSI University of Medicine and Health Sciences, Dublin, Ireland; 2 FutureNeuro SFI Research Centre, RCSI University of Medicine and Health Sciences, Dublin, Ireland

## Abstract

A central challenge in polygenic risk prediction is measuring and controlling for confounding due to population stratification. Standard approaches include adjusting for leading principal components (PCs) of genetic variation and using linear mixed models in genome-wide association studies (GWAS). Evidence of adequate control is typically inferred from reductions in the linkage disequilibrium score regression (LDSC) intercept and the preservation of polygenic risk score (PRS) performance in within-sibling settings. Here, we investigate how the number of PCs included during GWAS/PRS construction relates to the loss of predictive performance when moving from population-based analyses to that of discordant sibling pairs (within-sibling attenuation). This design detects confounding arising from environmental and genetic background effects correlated with population structure. Analyses were performed in the self-identified White subset of the UK Biobank (UKB) for coronary artery disease, type 2 diabetes, breast cancer, and prostate cancer, with educational attainment included as a comparison trait as it is known to exhibit substantial within-sibling attenuation. We find that increasing the number of PCs does not consistently reduce within-sibling attenuation across traits. Furthermore, reductions in attenuation do not closely correspond to decreases in the LDSC intercept, and mixed model–based methods offer little additional improvement in prediction or attenuation. Overall, our results suggest that confounding targeted by PC-adjustment and linear mixed models is either minimal in the UKB, or remains inadequately captured by current methods, reflecting limitations as to how much standard population structure correction improves the causal validity of PRS in the UKB.

**Supplementary Information:**

The online version contains supplementary material available at 10.1007/s00439-026-02852-3.

## Introduction

Genome-wide association studies (GWAS) have been performed on many human complex traits and diseases to associate single-nucleotide polymorphisms (SNPs) with phenotypes of interest (Visscher et al. 2017). The summary statistics of a GWAS include an effect size and P-value for each examined SNP. These summary statistics can be used to compute a polygenic risk score (PRS) in an external cohort by weighting each individual’s allele dosages by the estimated SNP effects and summing across variants (Choi et al. 2020). The potential ability of these PRSs to explain a large part of population-level variation of human disorders offers an important opportunity for personalized medicine. A polygenic score developed for coronary artery disease (CAD) demonstrates this potential. It identified individuals in the tail end of the risk score distribution that are at a three-fold higher risk of CAD than the general population, where this level of increased risk is comparable to that observed in individuals with monogenic forms of CAD (Khera et al. 2018). Thus, those individuals at high risk may be offered prophylactic treatment options, such as statins in the case of CAD, or preventative lifestyle interventions for other conditions such as Type 2 Diabetes (T2D) (Lewis and Green 2021; Torkamani et al. 2018). PRS have also shown to be substantial additional modifiers of disease risk or severity for several monogenic diseases such as diabetes or kidney disease (Khan et al. 2023; Murray Leech et al. 2025). Even in the cases where individual-level prediction is not accurate enough to justify prophylactic treatment, PRS could be an efficient and cost-effective population-level tool for choosing earlier ages of screening for disease, such as in the case of breast and prostate cancer (Mavaddat et al. 2019; McHugh et al. 2025). Therefore, there is much interest in the use of polygenic scores as a central tool in precision medicine.

One of the major challenges when associating a SNP with any complex phenotype is in determining the source of the association. An observed association between a SNP and a phenotype can be attributed to: direct genetic effects; indirect genetic effects (including parental and sibling effects); and confounding effects (Young et al. 2019). Direct genetic effects are causal relationships between that variant and the trait of interest, for example, a SNP’s direct impact on gene expression that raises or lowers disease risk. Indirect genetic effects are the effects of others’ genotypes on an individual’s phenotype, operating through the environment they create or modify. For example, a parental allele that increases the parent’s own smoking behaviour can raise the child’s exposure to pro-smoking norms, increasing the child’s propensity to smoke. This reflects an indirect genetic effect via the parental environment, beyond any direct effect of the child’s own genotype. Confounding effects are spurious SNP–phenotype associations arising not from causal pathways of the SNP on the phenotype, but instead because the genotype is correlated with other variables that influence the phenotype. Confounding effects can be attributed to three primary sources: environment; genetic background; and assortative mating (Young et al. 2019).

Environmental and genetic background confounding effects are often mediated through population structure in the data (Vilhjálmsson and Nordborg 2013; Barton et al. 2019). Ideally, the SNP effect size estimates included in a PRS would be reflective of causal genetic effects, but if this population structure is not properly accounted for, confounding may remain. Therefore, developing techniques to measure and reduce confounding has been a central task in the genomic prediction literature, with debate still remaining on how best to detect and minimize these unwanted effects (Price et al. 2006; Abegaz et al. 2019; Sul et al. 2018; Young 2024).

Perhaps the most commonly employed method for dealing with population structure has been the inclusion of principal components (PCs) of variation as covariates in GWAS. Principal component analysis (PCA) is a dimensionality reduction technique that generates orthogonal (i.e. linearly uncorrelated) components, each representing a distinct axis of genetic variation. In PCA, each successive PC explains progressively less variance than the previous.

The first PCs of a genomic SNP dataset often capture broad ancestry differences in the population, which can be used to control for confounding arising from population structure (Price et al. 2006; Novembre et al. 2008; Abegaz et al. 2019). In the context of the UK Biobank, prior studies suggest that the first 16 PCs are sufficient for this purpose. These PCs have been used for several GWAS and PRS conducted on this dataset in the wider literature (Privé et al. 2022; Hou et al. 2024; Gilchrist et al. 2025). Although PC-based methods may be sufficient to control for genetic background confounding and environmental confounding correlated with ancestry, it is not thought to contribute to removing associations driven by indirect genetic effects or assortative mating (Guan et al. 2025; Lee et al. 2018).

The addition of mixed model approaches to GWAS has now become common (Price et al. 2010; Yang et al. 2014). These methods employ a linear mixed model (LMM) and are commonly referred to as mixed-linear models of association (MLMAs). In this procedure, a genetic relatedness matrix (GRM) is constructed and is used to model the covariance structure of the random effect in the LMM. MLMA approaches are thought to better control for population stratification and cryptic relatedness than PC-adjustment alone. The MLMA approach may also provide some control for confounding due to assortative mating (Lee et al. 2018). Extensions appropriate for binary phenotypes through the use of generalized linear mixed models (GLMMs) have also been developed (Jiang et al. 2021).

Determining the success of methods that attempt to control for confounding in GWAS is challenging. The genomic inflation factor (λ) is a measure of global P-value inflation in the summary statistics outputted from a GWAS (Devlin and Roeder 1999; Reich and Goldstein 2001; Zheng et al. 2006; Yang et al. 2011b). When λ is significantly above one (i.e. P-values are systematically lower than the expectation under the null) this suggests that population stratification may not have been successfully corrected for, or that the trait is highly polygenic (Bulik-Sullivan et al. 2015). The intercept and ratio produced by linkage disequilibrium score regression (LDSC) is thought to then distinguish confounding signal from that of true polygenicity (Bulik-Sullivan et al. 2015; Alexander and Curtis 2020). The LDSC intercept provides an estimate of the absolute component of test statistic inflation that is consistent with confounding (with values close to 1 indicating minimal confounding), and the ratio value expresses this same quantity relative to the total inflation in the data, i.e. the proportion of inflation attributable to confounding rather than polygenic signal (with values ideally close to zero). Despite many such control and measurement techniques, some authors argue that the current methods may not yet be sufficient to truly correct for all the environmental and genetic background confounding present in GWAS effect size estimates (Berg et al. 2019; Young et al. 2019; Young 2024). As discussed above, much debate has surrounded the causal nature of the genetic effects estimated from GWAS and included in PRS models (Barton et al. 2019; Wray et al. 2013; Kaplan and Fullerton 2022; Vilhjálmsson and Nordborg 2013). This has been compounded by the fact that PRS models are often constructed with SNPs which fall well below the canonical genome-wide significance threshold, plausibly increasing the risk that genetic background and environmental confounding effects will be exploited during prediction (Choi et al. 2020; Janssens 2019).

One method that has been proposed as important to validating the causal nature of a given polygenic risk score has been the within-sibling study design. PRS trained on population data are expected to show reduced discrimination in within-sibling tests i.e. their predictive ability has been attenuated. This attenuation occurs for two principal reasons: (i) siblings share much of their SNP profile, which reduces PRS variation within families; and (ii) the within-sibling design removes signal due to population stratification, assortative mating, and most indirect genetic effects (with the exception of some sibling indirect effects) (Howe et al. 2022; Selzam et al. 2019; Lello et al. 2020). The degree to which a PRS discriminates between siblings, as compared to non-relatives in a population sample, is therefore thought to be informative about the proportion of the given PRS reflecting direct genetic effects (Young 2024; Veller and Coop 2024). Some indirect effects can remain even in these designs, and so caution is warranted when ascribing full causality to predictive power between siblings (Fletcher et al 2024).

Even when perfect control of genetic and environmental confounding is achieved when building a PRS, residual attenuation is still expected from assortative mating and indirect effects, and from the aforementioned reduced genetic variation among family members.

As predictive discrimination between siblings is in part due to the influence of causal genetic effects, measuring changes in predictive attenuation during population stratification control is seen as a valuable approach. PCA- and LMM-based methods target the genetic background and environmental component of confounding only. Therefore, if such confounding is a significant source of population-level performance, applying such adjustments should reduce the predictive attenuation observed when moving to a within-sibling setting. If attenuation does not reduce after adjustment, it can be inferred that this form of confounding was minimally important for the PRS, or that it is persisting in forms not well-captured by standard PCs and LMMs.

The approach taken in this paper follows from the work of Lello et al. who have previously developed polygenic risk scores from the UK Biobank and validated them in a within-sibling cohort (Lello et al. 2020). The authors found that a significant amount of polygenic predictive power remains when moving from a population-level cohort to a within-sibling setting. However, the authors did not make use of principal components or mixed models in the development of their PRS on the basis that their top PCs do not explain a substantial amount of phenotypic variation in the context of human height (Lello et al. 2018).

Extending this previous work, our study systematically evaluates how including principal components and using mixed models during GWAS and PRS construction affects within-sibling predictive attenuation across multiple complex traits. This design isolates attenuation attributable to population structure and ancestry-correlated confounding. Reductions in predictive attenuation observed with PC- and LMM-based adjustments likely reflect improved control of these factors, whereas remaining attenuation could stem from residual background or environmental confounding not captured by these methods, as well as from sources such as shared genetics, assortative mating, and indirect genetic effects. As improvements in prediction with stratification control should reflect a reduced contribution from confounding effects, the overall aim was to assess whether the commonly used methods for controlling for stratification appear to meaningfully impact the causal validity of PRS built using the UK Biobank.

## Materials and methods

### Study approach

We performed GWAS in the UK Biobank and constructed PRS to compare predictive performance between two types of disease-discordant pairs: unrelated case–control pairs and sibling case–control pairs. As principal components must be included as covariates at the GWAS stage, separate GWAS were performed for each level of PC-adjustment, generating distinct sets of summary statistics for downstream PRS construction. We assessed attenuation in classification accuracy when moving from population-level prediction to within-sibling prediction, and examined how varying the population structure adjustment strategy and SNP-set sizes influenced this attenuation.

### Phenotype selection

The study focused on the prediction of four common disorders for which sample sizes were relatively high and the potential for PRS in terms of clinical utility is high, namely: CAD, T2D, breast cancer and prostate cancer. Phenotype definitions followed those used in Lello et al. (2020) with the exception of educational attainment (EA) which was converted to a pseudo-binary phenotype in order to facilitate comparisons in PRS performance and attenuation (Lello et al. 2020). Dichotomizing educational attainment in this manner sacrifices information and interpretability and we employ it only to benchmark comparisons with the binary disease outcomes in terms of confounding. The precise encoding of each phenotype can be found in Supplementary note 1.

### Population selection and genotype quality control

As per Lello et al., all individuals with ethnic background codes 1, 1001, 1002, or 1003 (i.e. self-reported “White” individuals) were included in the GWAS and PRS analyses (Lello et al. 2020). The following quality control (QC) measures were implemented using PLINK1.9: minor allele frequency (MAF) threshold of 0.1%, SNP missingness filter of 3%, and individual missingness filter of 3% (Purcell et al. 2007). The extended human leukocyte antigen (HLA) region was excluded due to the complex nature of linkage disequilibrium in this region (Finucane et al 2015; Kennedy et al 2017). Sex chromosomes were also excluded due to SNP dosage differences between males and females.

### Discordant pair extraction

#### Siblings

Siblings were defined as those pairs which have an IBS0 > 0.0012 and kinship coefficient > 0.176. These criteria excluded monozygotic twins from this analysis. For each trait, all sibling pairs in which one individual was a case and the other a control were identified. Pairs were kept single-sex for prostate and breast cancer. Trios and higher-order relationships were not included in the analysis i.e. only the first pair were kept when more than one sibling pair was identified within a single family unit.

#### Non-siblings

After the creation of the discordant sibling test sets as described above, a KING relatedness threshold of ≥ 0.0884 was implemented to exclude closely related individuals from the post-QC dataset. Random case–control pairs were selected from the unrelated cohort, with sample sizes matched to the corresponding sibling test sets. For prostate and breast cancer, pairs were restricted to individuals of the same sex.

To match the age difference distribution observed in the sibling pairs, candidate non-sibling pairs were first stratified into equivalent age difference bins. Proportional sampling was then performed within these bins to replicate the sibling age difference distribution. The resulting set was trimmed or supplemented to match the exact number of sibling pairs, thus ensuring balanced age distributions and sample sizes between groups.

### Principal component analysis

Principal components were taken from UK Biobank data field 22009 and individuals with missing PC information were excluded (n = 14,227). The first 16 principal components have previously been stated to be sufficient to capture population structure in the UK Biobank (Privé et al. 2020).

### Association methods

#### Standard GWAS (GLM)

PLINK2’s --glm function was used to perform basic GWAS across the phenotypes (Chang et al. 2015). The discordant test sets made up of sibling and non-sibling pairs were excluded from the training GWAS so as not to bias results. To investigate the effect of including varying numbers of principal components of genetic variation, the top M PCs were included as covariates during association. Separate GWAS were therefore performed for each value of M, generating distinct summary statistics for PRS construction.

#### Mixed model GWAS

GCTA software was used to test for the added effect of performing a mixed model-based approach to controlling for population stratification (Yang et al. 2011a). Although MLMA approaches may capture the same effects as PCA, both approaches are generally used in combination, and this was the approach taken here (Zhang and Pan 2015; Zeng et al 2018). The post-QC SNPs were pruned using PLINK1.9 for construction of the GRMs with the following parameters: window-size of 100 kb, step-size of 5 bp and squared correlation threshold of 0.5 (Purcell et al. 2007). The HLA region was excluded during pruning. GRMs were constructed using GCTA version 1.92.3 on the resultant set of 111,226 SNPs. A sparsity threshold of 0.05 was then applied to the GRM. A GCTA 1.94.4 generalized linear mixed model association analysis (fastGWA-GLMM) was then performed on each trait with the full set of 16 principal components of variation included as covariates (Jiang et al. 2021).

### Clumping

SNPs were clumped using PLINK1.9 (Purcell et al. 2007). The LD threshold for clumping was set to 0.1, the distance threshold to 500 kb, and the index SNP significance threshold to 0.5.

#### LD-score regression

LD-Score Regression (LDSR) was implemented to distinguish test statistic inflation due to polygenicity from that of confounding (Bulik-Sullivan et al. 2015; Alexander and Curtis 2020). The regression intercept was estimated using the ldsr software v.1.01. Reference linkage disequilibrium (LD) scores and weights specific to those of European ancestries from the 1000 Genomes Project reference panel were used for this analysis (Siva 2008). The HLA region was excluded from the LD score reference files due to the complexity of linkage disequilibrium in the region.

#### Polygenic score construction

Polygenic scores were constructed for the discordant test set made up of sibling and non-sibling pairs with the top clumped SNPs ranked by GWAS P-value and using the --score function of PLINK2 (Chang et al. 2015). To standardize PRS construction across phenotypes, we fixed the number of included SNPs at 1000, 10,000, or 100,000 rather than applying a fixed P-value threshold. The raw scores were regressed on the top M principal components of genetic variation. The resulting residuals were used as the predictor variable for the phenotype of interest.

### Classification

The outcome variable for classification of discordant pairs was defined as the proportion of pairs in which the positive-class individual had the higher polygenic score (with ties excluded) (Lello et al. 2020).

This metric is conceptually similar to the area under the receiver operating characteristic curve (AUC) which represents the probability of correctly assigning a higher score to the case in a randomly selected discordant pair (Hanley and McNeil 1982). A major difference between the AUC and the classification metric used here is that the AUC represents an overall probability from all possible randomly selected discordant pairs in the population. The metric used here is based on a specific pre-defined and non-random set of pairs composed of the siblings and non-siblings.

Attenuation in performance was defined as the proportional reduction in above-chance classification accuracy when moving from unrelated pairs to siblings. An attenuation of 0% indicates no loss i.e. the PRS performed equally well in siblings and non-siblings. 100% attenuation corresponds to a complete loss of predictive power in the within-sibling setting. Negative attenuation values can arise in the rarer instance where there is a greater predictive power in the sibling pairs as compared to the non-sibling pairs.

### Statistical analysis

A generalized linear mixed model (GLMM) was fit to investigate the overall effects of pair type, adjustment method, phenotype, and SNP-set size on classification accuracy. Three adjustment methods were compared: 0-PC, 16-PC, and GLMM + 16-PC. An interaction term between method and pair type was included to assess whether the performance of the methods differed between sibling and non-sibling pairs. To account for repeated use of the same case–control pairs, a random intercept was included for each pair. The lme4 package was used to implement this GLMM using the BOBYQA optimizer (Bates et al. 2015; Powell et al. 2009).

## Results

### Datasets and traits analyzed

Analyses were conducted using the 2018 UK Biobank genotype release in the self-reported White ancestry subset. Principal component analysis (PCA) plots are shown in Supplementary Figures S1 and S2. We examined four disease traits for which polygenic risk scores have shown potential clinical utility (Sudlow et al. 2015; Bycroft et al. 2018). For the purposes of comparison, we examined the effects of controlling for population stratification on a binarized version of the educational attainment phenotype which has been well-established as having issues with confounding and within-family attenuation (Lee et al. 2018; Selzam et al. 2019; Lello et al. 2020). The relative importance of contributing factors to this attenuation is not currently settled, with some suggestion that population stratification may play a major role (Tan et al. 2024; Smith et al. 2025). Other authors have also found a large role for indirect genetic effects in this trait (Mostafavi et al. 2020; Howe et al. 2022).

Summary statistics from each GWAS were used to build PRS models used to classify between discordant pairs (consisting of one case and one control in each pair). Two test sets were evaluated, one consisting of sibling pairs and the other of population-level unrelated pairs (non-siblings). To account for the effects of different levels of polygenicity, three SNP-set sizes were examined during PRS construction (1 K, 10 K, and 100 K). The total sample size of each phenotype, and the discordant sibling and non-sibling test sets can be seen in Tables 1 and 2 respectively.

**Table 1:** Sample sizes in training GWAS for positive and negative phenotype classes in the UK Biobank

Phenotype	Positive	Negative
Coronary artery disease	45,810	367,385
Type 2 diabetes	30,936	385,931
Breast cancer	9548	217,739
Prostate cancer	4075	190,692
Educational attainment	64,941	347,166

**Table 2:** The number of individuals in discordant sibling test sets from the UK Biobank

Phenotype	Number of siblings
Coronary artery disease	7512
Type 2 diabetes	5024
Breast cancer	1196
Prostate cancer	294
Educational attainment	8280

Additional test sets made up of an equal number of unrelated population-level pairs were generated

The primary outcome was the attenuation in predictive performance observed when moving from population-level to within-sibling prediction.

### Baseline PRS accuracy and attenuation

To establish baseline performance metrics and quantify the degree of confounding present prior to population structure adjustment, we first constructed PRSs using standard GLM-based GWAS without controlling for population stratification (i.e. no PCs were included as covariates). The predictive accuracy of these PRSs in both the population-level and within-sibling settings is presented in Table 3. Overall, predictive accuracy was modest across all traits, with no PRS exceeding 65% classification accuracy in the population-level setting. As expected, we observed reduced accuracy among discordant sibling pairs compared to unrelated individuals across most trait-SNP set combinations, reflecting within-sibling attenuation. Educational attainment exhibited the largest attenuation, with the majority of classification accuracy lost when transitioning from the population-level to within-sibling setting, consistent with prior literature documenting substantial confounding in this trait (Lee et al. 2018; Selzam et al. 2019; Lello et al. 2020).

**Table 3:** Baseline classification accuracy and attenuation for standard GWAS-based PRS performance on sibling and population-level (non-sibling) discordant pairs in the UK Biobank

Phenotype	SNP-set size	Classification accuracy (%)		Attenuation (%)
		Non-sibling pairs	Sibling pairs	
Coronary artery disease	**1 K**	**57.29**	**53.78**	**48.1**
	10 K	56.58	55.40	17.9
	100 K	55.99	55.01	16.4
Type 2 diabetes	1 K	62.46	59.28	25.5
	**10 K**	**63.54**	**59.00**	**33.5**
	100 K	63.54	58.52	37.1
Breast cancer	**1 K**	**58.03**	**54.85**	**39.6**
	10 K	56.52	53.68	43.6
	100 K	55.02	54.01	20.1
Prostate cancer	**1 K**	**58.50**	**60.54**	**−24.0**
	10 K	57.82	55.78	26.1
	100 K	57.14	54.42	38.1
Educational attainment	1 K	58.33	51.93	76.8
	**10 K**	**59.76**	**53.48**	**64.3**
	100 K	59.69	53.14	67.6

No principal components were included as covariates in either the GWAS or PRS

Among the disease traits, attenuation was generally significant but more moderate in magnitude. A notable exception to this was that the 1000 SNP prostate cancer model showed negative attenuation, with the PRS demonstrating higher predictive accuracy in the sibling set than among unrelated individuals. This pattern was not observed for other SNP set sizes and may be reflective of stochastic variation.

We did not observe a consistent relationship between SNP set size and either predictive accuracy or attenuation magnitude. Specifically, larger SNP sets did not consistently yield improved prediction in the population-level setting, nor did they exhibit increased attenuation as might be expected if the inclusion of SNPs with lower P-values was more susceptible to capturing confounding effects. Based on performance in the population-level test set, we selected the best-performing SNP set size for each trait to carry forward in subsequent analyses examining the impact of PC adjustment (bolded in Table 3).

### Changes in the LDSC intercept with increasing PC inclusion

**Fig. 1:**
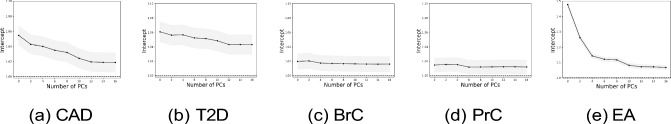
Changes in the LD score regression (LDSC) intercept with increasing inclusion of principal components (PCs) across phenotypes: coronary artery disease (CAD), type 2 diabetes (T2D), breast cancer (BrC), prostate cancer (PrC), and binarized educational attainment (EA). LDSC summary statistics were derived from GLM-based GWAS results. Shaded regions represent standard errors. The y-axis limit is higher for educational attainment due to greater overall inflation

To evaluate whether standard population structure adjustment methods effectively reduce confounding as traditionally measured, we examined the effect of including an increasing number of PCs on the LDSC intercept and ratio across all traits. The LDSC intercept results are presented in Fig. 1, with corresponding LDSC ratio trends shown in Supplementary Figure S4. The intercept decreased for most traits as the number of PCs included in the training GWAS increased, though the magnitude and pattern of reduction varied substantially across phenotypes.

Consistent with the baseline attenuation results, educational attainment exhibited the strongest response to PC-adjustment. Both the intercept and ratio were substantially elevated at baseline (no PCs included), indicating considerable confounding. While subsequent inclusion of PCs produced marked reductions in both metrics, residual confounding likely remained even when all sixteen principal components were included as covariates (Wang et al. 2023). Coronary artery disease and type 2 diabetes demonstrated moderate baseline evidence of confounding, with intercepts modestly elevated above 1.0. Both traits showed downward trends in the intercept as more PCs were included, though the absolute magnitude of change was not extreme. Notably, after PC correction with the full complement of 16 PCs from data field 22009, both traits achieved LDSC ratios below 1.1, falling under a rule-of-thumb threshold for minor confounding (Bulik-Sullivan Lab 2025). Breast and prostate cancer exhibited low absolute levels of confounding at baseline. The LDSC ratios for these traits were more moderate but associated with large standard errors, possibly reflecting sample size limitations for these two phenotypes. Finally, similar patterns as these trends were observed for the genomic inflation factor λ across all traits (Supplementary Figure S3).

### PRS accuracy across levels of PC inclusion

To assess whether reductions in test statistic inflation translate to changes in predictive performance, we constructed PRSs for each phenotype across all levels of PC inclusion, with PCs included as covariates in both the training GWAS and during PRS prediction.

**Fig. 2:**
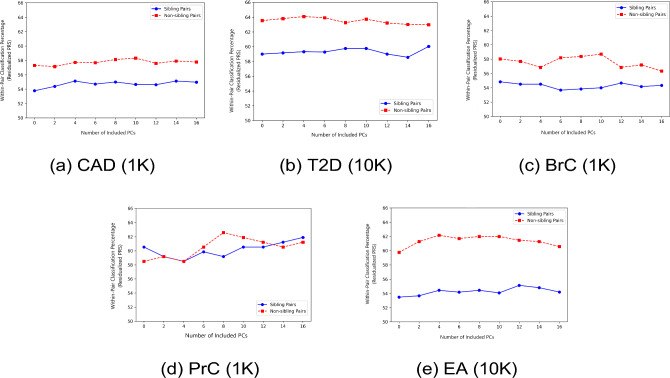
PRS classification accuracy across levels of PC inclusion for sibling and non-sibling discordant pairs, shown for the best-performing SNP-set size per phenotype

Results for the best-performing SNP set size for each trait are presented in Fig. 2. Among unrelated individuals, we observed no consistent monotonic relationship between the number of included PCs and predictive accuracy, though modest variation across PC levels was evident. Among discordant sibling pairs, there was a slight overall trend toward improved performance with increasing PC inclusion, though this pattern was not universal across all traits.

With respect to phenotype-specific patterns, the largest variation in accuracy was observed for breast and prostate cancer. Across both sibling and population pairs, the first six PCs generally increased the accuracy for the EA phenotype, while only modest changes in magnitude were observed for CAD and T2D. When examining the full range of SNP set sizes, the absence of a consistent trend in either unrelated individuals or sibling pairs became even more apparent (Supplementary Figures S5–S9).

The principal components used in these analyses were computed across the entire UK Biobank cohort, including individuals of more diverse ancestry than those included in our ancestry-filtered GWAS. To assess whether this might diminish the effectiveness of PC-adjustment for our analysis cohort, we recomputed PCs using only individuals from the post-QC, self-described White subset. When these cohort-specific PCs were used in GWAS and PRS construction, they did not substantially alter predictive accuracy for any of the disease traits examined (Supplementary Tables S2 and S3).

Whilst overall predictive accuracy is informative, the primary outcome of interest for evaluating confounding is not absolute performance. PRS accuracy may reflect both causal genetic effects and confounding, whereas within-sibling attenuation directly quantifies the degree to which population-level associations fail to replicate in a setting that controls for both genetic background and environmental confounding.

### Predictive attenuation across levels of PC inclusion

We next examined whether PC-adjustment reduces within-sibling attenuation. Reductions in attenuation when transitioning from the population-level to within-sibling setting would indicate that PC-adjustment successfully controls for environmental and genetic background confounding. The experimental design here allows us to isolate the specific effects of environmental and genetic background confounding that PC-adjustment is intended to target. Whilst some persistent attenuation is expected due to shared genetics between siblings and the other sources of confounding not addressed by PC-adjustment, reductions in attenuation with increasing PC inclusion can provide evidence that standard population structure adjustment methods are successfully mitigating the confounding they are specifically designed to control.

**Fig. 3:**
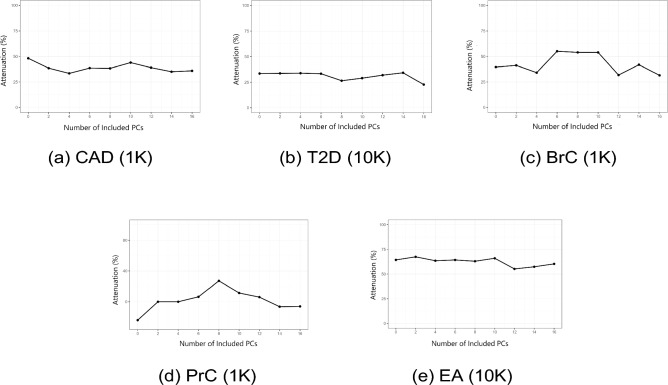
Attenuation in PRS predictive performance between sibling and non-sibling discordant pairs across levels of PC inclusion, shown for the best-performing SNP-set size per phenotype

Attenuation results for each trait are presented in Fig. 3. Across all traits, we did not observe a consistent decrease in attenuation as more PCs were included. A weak trend toward reduced attenuation may be present in some cases, although the relationship was neither monotonic nor uniform. Notably, attenuation sometimes increased or decreased in a non-systematic manner depending on the specific number of included PCs, suggesting that the optimal number of PCs for minimizing attenuation is not predictable in advance of within-sibling validation, or through observation of the LDSC intercept. This lack of consistent pattern was further confirmed when examining all SNP set sizes (Supplementary Figure S10).

Educational attainment exhibited persistently high attenuation across all levels of PC inclusion, despite the substantial reduction in LDSC intercept documented above. Similarly, for coronary artery disease and type 2 diabetes, the decreases in LDSC intercept observed with increasing PC inclusion were not accompanied by corresponding reductions in within-sibling attenuation. These findings indicate that strong-to-moderate reductions in confounding as measured by the LDSC intercept do not translate to meaningful improvements in within-sibling predictive validity. This suggests some limitations in using test statistic inflation metrics as proxies for the causal validity of polygenic risk scores.

Our results are consistent with prior work suggesting that population structure (as captured by PCs) may contribute only modestly to PRS performance for some traits in the UK Biobank. For example, Lello et al. showed for height that regressing the phenotype on principal components yields low explanatory power, implying limited variance attributable to PC-defined structure (Lello et al. 2018). Similarly, although several PCs were significantly associated with each phenotype in our data, we did not observe a clear relationship between the significant PCs and specific changes in predictive performance or within-sibling attenuation, despite the downward trends in overall inflation mediated by PC-adjustment (Supplementary Table S1).

### Additional effects of mixed model GWAS

To investigate whether GRM-based mixed models provide additional control over confounding beyond PC-adjustment alone, we performed GWAS using fastGWA-GLMM with the full set of 16 PCs included as covariates. We compared the resulting PRSs to those derived from standard GLM-based GWAS with either 0 PCs or 16 PCs.

Results for the best-performing SNP set size for each trait are presented in Table 4. As already shown in the previous findings, inclusion of the full set of PCs may have produced slight improvements in accuracy and reductions in attenuation relative to models with no PC adjustment. However, the addition of mixed model correction provided no apparent benefit beyond that achieved through PC inclusion alone. Within-sibling attenuation was nearly identical between the 16-PC GLM and GLMM + 16-PC approaches across most traits examined.

**Table 4:** Classification accuracy and within-sibling attenuation for PRS derived from GWAS across population structure adjustment methods

Phenotype	Method	Classification accuracy (%)		Attenuation (%)
		Non-sibling pairs	Sibling pairs	
Coronary artery disease	0-PC	57.29	53.78	48.1
(1 K)	16-PC	57.77	54.98	35.9
	GLMM + 16-PC	57.72	54.98	35.5
Type 2 diabetes	0-PC	63.54	59.00	33.5
(10 K)	16-PC	62.98	60.03	22.7
	GLMM + 16-PC	63.02	59.83	24.5
Breast cancer	0-PC	58.03	54.85	39.6
(1 K)	16-PC	56.35	54.35	31.5
	GLMM + 16-PC	55.69	54.85	14.8
Prostate cancer	0-PC	58.50	60.54	−24.0
(1 K)	16-PC	61.22	61.90	−6.1
	GLMM + 16-PC	59.18	61.22	−22.2
Educational attainment	0-PC	59.76	53.48	64.3
(10 K)	16-PC	60.58	54.20	60.3
	GLMM + 16-PC	60.72	54.18	61.0

Results are shown for sibling and non-sibling discordant pairs from the UK Biobank

For each phenotype, rows correspond to an unadjusted GLM-based GWAS (0-PC), a GLM-based GWAS including 16 PCs (16-PC) from data field 22,009, and a mixed model-based GWAS including 16 PCs (GLMM + 16-PC)

This pattern was also largely consistent across all SNP set sizes (Tables 3, S5, and S6). Furthermore, the GLMM approach did not meaningfully reduce the genomic inflation factor λ beyond the level achieved with 16 PCs in a standard GLM framework (Supplementary Table S4). Taken together, these results suggest that in large and relatively homogeneous cohorts such as the UK Biobank, mixed model methods do not offer substantive improvements over PC-adjustment for controlling confounding effects attributable to population stratification.

To more formally assess the factors influencing classification accuracy, we fit a generalized linear mixed model (GLMM) with phenotype, SNP-set size, pair type (sibling vs. non-sibling), and adjustment method (0-PC, 16-PC, and GLMM + 16-PC) as fixed effects. This analysis allowed the relative importance of each factor to be evaluated while accounting for repeated measurements across traits and models.

Phenotype emerged as the strongest predictor of classification accuracy (χ^2^ = 114, p < 1 × 10^−15^). Pair type was also strongly associated with accuracy (χ^2^ = 80.1, 14 p < 1 × 10^−15^), reflecting the substantial reduction in predictive performance observed when moving from population-level to within-sibling comparisons. SNP-set size had a more modest but still statistically significant effect (χ^2^ = 21.6, p = 2 × 10^−5^).

In contrast, population structure adjustment method explained relatively little variation in predictive accuracy overall (χ^2^ = 9.6, p = 0.008), with both the 16-PC and GLMM + 16-PC approaches yielding only slight improvements relative to the unadjusted model. Importantly, the interaction between adjustment method and pair type was not significant (χ^2^ = 0.008, p = 0.996), indicating that all three methods exhibited comparable levels of attenuation when transitioning from unrelated individuals to sibling pairs.

Together, these results suggest that more sophisticated population structure correction strategies do not substantially improve within-sibling transferability of PRS beyond standard PC adjustment in this dataset, and that reductions in population-level test-statistic inflation do not necessarily translate into gains in causal predictive validity.

## Discussion

Our study design enabled us to assess how effectively principal component adjustment and mixed models reduced within-sibling PRS attenuation attributable to genetic background and environmental confounding. Because predictive power in sibling-based analyses generally reflects direct genetic effects to a greater degree than population-level analyses, this framework allowed us to evaluate whether standard population stratification controls improve the causal validity of PRS models in the UK Biobank.

Several expectations regarding population stratification control in PRS development did not consistently hold across traits in this analysis. In particular, increasing the number of principal components included during GWAS and PRS construction did not reliably reduce within-sibling attenuation of predictive performance. Our formal statistical analysis confirmed that the PC-adjustment and mixed model approaches exhibited similar attenuation to the baseline approach when moving from unrelated to sibling pairs. Whilst a general reduction was observed in the genomic inflation factor λ and LDSC intercept with the inclusion of additional PCs, this did not consistently translate into improvements in the within-sibling prediction task. These findings suggest that reductions in test statistic inflation should not be interpreted as direct evidence of significantly improved causal validity of PRS models.

It is common to use both PCA and mixed model approaches in GWAS applications and some debate remains with regards to how complementary the two methods may be (Zhang and Pan 2015; Yao and Ochoa 2023). In this study, the use of linear mixed models for the generation of GWAS summary statistics did not significantly reduce either λ or attenuation beyond that of PC-control alone. We therefore do not advise against the use of both approaches simultaneously, but do acknowledge there may be redundancy in the information being captured.

For several traits, breast and prostate cancer in particular, the effects of attempting stratification control appeared minimal regardless of the number of PCs included or the use of LMMs. This potentially reflects low baseline levels of confounding in these traits, or limited power to detect and minimize its effects. Even for educational attainment, which is thought to have substantial vulnerability to confounding, the success of PC or mixed model-based correction in fully accounting for stratification was questionable. Whilst the LDSC intercept and λ decreased with additional PCs as expected, a concomitant reduction in attenuation was not observed. Variation in SNP-set size also had little consistent effect on within-sibling attenuation or population-level performance across traits. This suggests that increased polygenicity does not necessarily exacerbate confounding effects or alter the effectiveness of stratification control in this context.

Even after substantial attempts at stratification control, attenuation in prediction remained substantial in some traits. This was expected, as within-sibling attenuation can arise from several sources, including genomic sharing between siblings, indirect parental effects, and assortative mating (Lee et al. 2018; Kong et al. 2018; Howe et al. 2022). These factors are expected to contribute mainly to the baseline level of attenuation seen across our experimental design. Our primary focus, however, was not the total level of attenuation itself, but whether attenuation changed systematically across PC-adjustment levels. Reductions in attenuation following stratification control would be consistent with a component attributable to genetic background or environmental confounding captured by these methods. The remaining attenuation after such corrections could reflect shared genetics, indirect genetic effects, assortative mating, residual confounding, or other sources that our experimental design cannot not distinguish between.

The relationship between test statistic inflation and within-sibling attenuation varied substantially across traits in ways that provide insight into the nature of the confounding present. For the binarized educational attainment phenotype, both the LDSC intercept and ratio were substantially elevated at baseline, indicating considerable confounding. This served as a useful benchmark to compare the disease phenotypes with. PC-adjustment produced marked reductions in both values, yet within-sibling attenuation remained persistently high. This dissociation suggests that the confounding captured by these metrics may not be especially relevant in terms of trait prediction. For coronary artery disease and type 2 diabetes, baseline LDSC metrics were moderately elevated. PC-adjustment reduced both metrics, with ratios falling to acceptable levels (below 1.1) after inclusion of 16 PCs, though the overall magnitude of this reduction was not particularly large. Despite some improvements in test statistic inflation, a corresponding reduction in within-sibling attenuation was not observed. Attenuation for these traits did fluctuate depending on the specific number of PCs included, but no general downward trend was observed as more PCs were added. This lack of systematic relationship between PC inclusion and attenuation, despite measurable reductions in LDSC metrics, again suggests that the confounding captured and reduced by standard test statistic inflation measures may not correspond to the confounding affecting within-sibling prediction validity. Overall, however, LDSC statistic improvements were modest relative to educational attainment, limiting their potential impact on within-sibling attenuation.

Taken together, these findings suggest that current best practices, such as adjusting for a pre-defined fixed number of PCs, may not generalize well across phenotypes. Many GWAS and PRS models derived from the UK Biobank resource employ these or similar adjustment methods as default (Privé et al. 2022; Hou et al. 2024; Gilchrist et al. 2025). The present results, however, raise the question of whether these corrections are consistently useful in this cohort. It remains unclear whether the limited influence of stratification control on attenuation reflects genuinely minimal confounding within the UK Biobank or, conversely, confounding that persists in forms not adequately addressed by conventional methods. If the former is true, routine application of these corrections may add complexity without substantive benefit. If the latter is true, more sophisticated approaches will be required to capture subtle or residual population structure. Although we cannot determine definitively whether any remaining attenuation after adjustment is due to environmental or genetic background confounding, this possibility cannot be excluded and must still be considered.

Regardless of the underlying mechanism behind predictive power, be that genetically causal or not, polygenic risk scores that successfully discriminate higher-risk individuals at a population-level may still serve a useful function in clinical and public health contexts. Debate remains as to whether or not a lack of full causal interpretability necessarily precludes implementation of PRS as a screening or risk stratification tool (Abdellaoui and Verweij 2021; Barton et al. 2019; Kaplan and Fullerton 2022; Wray et al. 2013). Although this study focused only on linear polygenic risk scores, the question of establishing causality may increase in importance if the field of genomic prediction shifts toward reliance on “black-box” machine-learning models such as neural networks (Bellot et al. 2018; Kelly and McLaughlin 2024). Non-linear models could exploit complex confounding patterns that cannot be adequately addressed through linear approaches such as PCA. For this reason, focus on the development of robust validation frameworks for assessing the causal interpretation of PRS may increase in the years ahead. Ultimately, GWAS associations between SNPs and phenotypes are observational, and the experimental evidence needed to establish causality for the variants included in polygenic scores is likely to remain lacking. We recommend that all forms of confounding control be considered carefully when building a PRS model. The number of PCs included should be chosen with care, and sibling validation can be an important tool in this process. The UKB is an important resource for sibling validation, but other phenotype-specific datasets should also be used where available. Estimation of the genomic inflation factor and the LDSC intercept also remains important, and further efforts to ensure that PRS exploit only truly direct genetic effects should be expanded.

Several limitations should be considered when interpreting these findings. Firstly, the number of traits included in this analysis was small and the sample sizes of the test sets were relatively modest, especially for the two cancer phenotypes. The size of currently available within-family cohorts is limited, however, there do exist external independent datasets that could be used to potentially validate some of these findings (Howe et al. 2022). Sibling trios (or larger set sizes) could be used to increase the power of analysis, but their sample sizes were limited in the UKB. We would therefore encourage the growth of datasets that have detailed genotype and phenotype information on large sibling pedigrees. The sample sizes for SNP association were large, but still generally smaller than those used for some modern GWAS. This sample size and the relatively homogenous nature of the UKB might explain the overall modest performance of the PRS generated here, although the focus of the study was in changes in accuracy with stratification control, rather than the overall magnitude.

Secondly, sibling indirect effects may marginally affect within-sibling PRS performance, meaning that predictive signal between siblings may not solely reflect strictly proximally biologically causal pathways. However, the present design specifically evaluated improvements in causal validity associated with population stratification control methods, which are not expected to mitigate sibling indirect effects. As such, these effects are unlikely to substantially influence the interpretation of our results.

Thirdly, the results shown here only apply to those of self-declared White ancestry and present in the UK Biobank. The nature of the recruitment processes and relatively small geographic catchment area might limit the extent to which environmental and genetic background confounding is an issue in the GWAS being conducted on this data. Analyses on other populations or more diverse cohorts could see much larger effects of stratification control, especially in those of admixed continental ancestry.

Lastly, we acknowledge that the results obtained may differ depending on additional phenotype-specific covariates being included in both the GWAS and PRS models. The focus of this analysis was on the specific effects of principal components as covariates but we also attempted control for sex and age, however, there may be additional effects not detected here when other covariates are included during association and model building.

In conclusion, attempting to control for population stratification on polygenic risk scores derived from UK Biobank data warrants careful consideration. The relationship between test statistic inflation, principal component or mixed model adjustment, and the within-sibling attenuation is complex and not readily predictable. No single correction heuristic appears sufficient to maximize predictive performance and aid in establishing causality across all the traits examined. We therefore recommend incorporating direct within-sibling validation, when possible, to empirically inform the selection of population stratification control measures being used during model construction.

## Supplementary Information

Below is the link to the electronic supplementary material.

Supplementary file1 (DOCX 1456 KB)

## Data Availability

This study was conducted using the UK Biobank resource under approved application 103770. Individual-level genotype and phenotype data from the UK Biobank are available upon application to the UK Biobank. Data and scripts relevant to this work are available from: https://github.com/ciaranoceallaigh96/sibling_validation_ukbb.

## References

[CR1] Abdellaoui A, Verweij KJH (2021) Dissecting polygenic signals from genome-wide association studies on human behaviour. Nat Hum Behav. 10.1038/s41562-021-01110-y 33986517 10.1038/s41562-021-01110-y

[CR2] Abegaz F, Chaichoompu K, G´enin E et al (2019) Principals about principal components in statistical genetics. Brief Bioinf 20(6):2200–2216. 10.1093/bib/bby081 10.1093/bib/bby081 30219892

[CR3] Alexander M, Curtis D (2020) Ld scores are associated with differences in allele frequencies between populations but ld score regression can still distinguish confounding from polygenicity. Annal Hum Genet 84:412–416. 10.1111/ahg.12370 10.1111/ahg.12370 31925776

[CR4] Barton N, Hermisson J, Nordborg M (2019) Population genetics: why structure matters. Elife. 10.7554/eLife.45380 30895925 10.7554/eLife.45380 PMC6428565

[CR5] Bates D, Machler M, Bolker B et al (2015) Fitting linear mixed-effects models using lme4. J Stat Softw 67(1):1–48. 10.18637/jss.v067.i0

[CR6] Bellot P, de Los Campos G, Perez-Enciso M (2018) Can deep learning improve genomic prediction of complex human traits? Genetics 210(3):809–819. 10.1534/genetics.118.301298 30171033 10.1534/genetics.118.301298 PMC6218236

[CR7] Berg JJ, Harpak A, Sinnott-Armstrong N et al (2019) Reduced signal for polygenic adaptation of height in UK biobank. Elife. 10.7554/eLife.39725 30895923 10.7554/eLife.39725 PMC6428572

[CR8] Bulik-Sullivan BK, Loh PR, Finucane HK et al (2015) LD Score regression distinguishes confounding from polygenicity in genome-wide association studies. Nat Genet 47(3):291–295. 10.1038/ng.3211 25642630 10.1038/ng.3211 PMC4495769

[CR9] Bulik-Sullivan Lab (2025) Heritability and genetic correlation. LDSC GitHub Wiki, URL https://github.com/bulik/ldsc/wiki/Heritability-and-Genetic-Correlation, Accessed: 2025–12–18

[CR10] Bycroft C, Freeman C, Petkova D et al (2018) The UK biobank resource with deep phenotyping and genomic data. Nature. 10.1038/s41586-018-0579-z 30305743 10.1038/s41586-018-0579-z PMC6786975

[CR11] Chang CC, Chow CC, Tellier LC et al (2015) Second-generation PLINK: rising to the challenge of larger and richer datasets. GigaScience 4(1):7. 10.1186/s13742-015-0047-8 25722852 10.1186/s13742-015-0047-8 PMC4342193

[CR12] Choi SW, Mak TSH, O’Reilly PF (2020) Tutorial: a guide to performing polygenic risk score analyses. Nat Protoc 15:2759–2772. 10.1038/s41596-020-0353-1 32709988 10.1038/s41596-020-0353-1 PMC7612115

[CR13] Devlin B, Roeder K (1999) Genomic control for association studies. Biometrics 55:997–1004. 10.1111/j.0006-341X.1999.00997.x 11315092 10.1111/j.0006-341x.1999.00997.x

[CR14] Finucane HK, Bulik-Sullivan B, Gusev A et al (2015) Partitioning heritability by functional annotation using genome-wide association summary statistics. Nat Genet 47(11):1228–1235. 10.1038/ng.3404 26414678 10.1038/ng.3404 PMC4626285

[CR15] Fletcher J, Wu Y, Li T, Lu Q (2024) Interpreting polygenic score effects in sibling analysis. PLoS ONE 19(2):e0282212 38358994 10.1371/journal.pone.0282212 PMC10868763

[CR17] Gilchrist L, Spargo TP, Green RE et al (2025) Depression symptom-specific genetic associations in clinically diagnosed and proxy case Alzheimer’s disease. Nat Mental Health 3:212–228. 10.1038/s44220-024-00369-0

[CR18] Guan J, Tan T, Nehzati SM et al (2025) Family-based genome-wide association study designs for increased power and robustness. Nat Genet 57(4):1044–1052 40065166 10.1038/s41588-025-02118-0 PMC11985344

[CR19] Hanley JA, McNeil BJ (1982) The meaning and use of the area under a receiver operating characteristic (roc) curve. Radiology 143:29–36. 10.1148/radiology.143.1.7063747 7063747 10.1148/radiology.143.1.7063747

[CR20] Hou K, Xu Z, Ding Y et al (2024) Calibrated prediction intervals for polygenic scores across diverse contexts. Nat Genet 56:1386–1396. 10.1038/s41588-024-01792-w 38886587 10.1038/s41588-024-01792-w PMC11465192

[CR21] Howe LJ, Nivard MG, Morris TT et al (2022) Within-sibship genome-wide association analyses decrease bias in estimates of direct genetic effects. Nat Genet 54:581–592. 10.1038/s41588-022-01062-7 35534559 10.1038/s41588-022-01062-7 PMC9110300

[CR22] Janssens ACJW (2019) Validity of polygenic risk scores: are we measuring what we think we are? Hum Mol Genet 28:R143–R150. 10.1093/hmg/ddz205 31504522 10.1093/hmg/ddz205 PMC7013150

[CR23] Jiang L, Zheng Z, Fang H et al (2021) A generalized linear mixed model association tool for biobank-scale data. Nat Genet 53:1616–1621. 10.1038/s41588-021-00954-4 34737426 10.1038/s41588-021-00954-4

[CR24] Kaplan JM, Fullerton SM (2022) Polygenic risk, population structure and ongoing difficulties with race in human genetics. Philos Trans R Soc B: Biol Sci 377(1852):20200427. 10.1098/rstb.2020.0427 10.1098/rstb.2020.0427 PMC9014185 35430888

[CR25] Kelly CM, McLaughlin RL (2024) Comparison of machine learning methods for genomic prediction of selected *Arabidopsis thaliana* traits. PLoS ONE 19:e0308962. 10.1371/journal.pone.0308962 39196916 10.1371/journal.pone.0308962 PMC11355539

[CR26] Kennedy AE, Ozbek U, Dorak MT (2017) What has GWAS done for HLA and disease associations? Int J Immunogenet 44:195–211. 10.1111/iji.12332 28877428 10.1111/iji.12332

[CR27] Khan A, Shang N, Nestor JG et al (2023) Polygenic risk alters the penetrance of monogenic kidney disease. Nat Commun 14(1):8318 38097619 10.1038/s41467-023-43878-9 PMC10721887

[CR28] Khera AV, Chaffin M, Aragam KG et al (2018) Genome-wide polygenic scores for common diseases identify individuals with risk equivalent to monogenic mutations. Nat Genet 50(9):1219–1224. 10.1038/s41588-018-0183-z 30104762 10.1038/s41588-018-0183-z PMC6128408

[CR29] Kong A, Thorleifsson G, Frigge ML et al (2018) The nature of nurture: effects of parental genotypes. Science 359:424–428. 10.1126/science.aan6877 29371463 10.1126/science.aan6877

[CR30] Lee JJ, Wedow R, Okbay A et al (2018) Gene discovery and polygenic prediction from a genome-wide association study of educational attainment in 1.1 million individuals. Nat Genet 50:1112–1121. 10.1038/s41588-018-0147-3 30038396 10.1038/s41588-018-0147-3 PMC6393768

[CR31] Lello L, Avery SG, Tellier L et al (2018) Accurate genomic prediction of human height. Genetics 210(2):477–497. 10.1534/genetics.118.301267 30150289 10.1534/genetics.118.301267 PMC6216598

[CR32] Lello L, Raben TG, Hsu SDH (2020) Sibling validation of polygenic risk scores and complex trait prediction. Sci Rep 10(1):13190. 10.1038/s41598-020-69927-7 32764582 10.1038/s41598-020-69927-7 PMC7411027

[CR33] Lewis ACF, Green RC (2021) Polygenic risk scores in the clinic: new perspectives needed on familiar ethical issues. Genome Med 13(1):14. 10.1186/s13073-021-00829-7 33509269 10.1186/s13073-021-00829-7 PMC7844961

[CR34] Mavaddat N, Michailidou K, Dennis J et al (2019) Polygenic risk scores for prediction of breast cancer and breast cancer subtypes. Am J Hum Genet 104(1):21–34 30554720 10.1016/j.ajhg.2018.11.002 PMC6323553

[CR35] McHugh JK, Bancroft EK, Saunders E et al (2025) Assessment of a polygenic risk score in screening for prostate cancer. N Engl J Med 392(14):1406–1417 40214032 10.1056/NEJMoa2407934 PMC7617604

[CR36] Mostafavi H, Harpak A, Agarwal I et al (2020) Variable prediction accuracy of polygenic scores within an ancestry group. Elife 9:12223. 10.7554/eLife.48376 10.7554/eLife.48376 PMC7067566 31999256

[CR37] Murray Leech J, Beaumont RN, Arni AM et al (2025) Common genetic variants modify disease risk and clinical presentation in monogenic diabetes. Nat Metab 7(9):1819–1829 40925988 10.1038/s42255-025-01372-0 PMC12460161

[CR38] Novembre J, Johnson T, Bryc K et al (2008) Genes mirror geography within Europe. Nature 456(7218):98–101. 10.1038/nature07331 18758442 10.1038/nature07331 PMC2735096

[CR39] Powell MJ et al (2009) The bobyqa algorithm for bound constrained optimization without derivatives. Cambridge NA Report NA2009/06, University of Cambridge, Cambridge 26:26–46

[CR40] Price AL, Patterson NJ, Plenge RM et al (2006) Principal components analysis corrects for stratification in genome-wide association studies. Nat Genet. 10.1038/ng1847 16862161 10.1038/ng1847

[CR41] Price AL, Zaitlen NA, Reich D et al (2010) New approaches to population stratification in genome-wide association studies. Nat Rev Genet 11:459–463. 10.1038/nrg2813 20548291 10.1038/nrg2813 PMC2975875

[CR42] Privé F, Luu K, Blum MGB et al (2020) Efficient toolkit implementing best practices for principal component analysis of population genetic data. Bioinformatics 36:4449–4457. 10.1093/bioinformatics/btaa520 32415959 10.1093/bioinformatics/btaa520 PMC7750941

[CR43] Privé F, Aschard H, Carmi S et al (2022) Portability of 245 polygenic scores when derived from the uk biobank and applied to 9 ancestry groups from the same cohort. Am J Hum Genet 109:12–23 34995502 10.1016/j.ajhg.2021.11.008 PMC8764121

[CR44] Purcell S, Neale B, Todd-Brown K et al (2007) PLINK: a tool set for whole-genome association and population-based linkage analyses. Am J Hum Genet 81(3):559–575. 10.1086/519795 17701901 10.1086/519795 PMC1950838

[CR45] Reich DE, Goldstein DB (2001) Detecting association in a case-control study while correcting for population stratification. Genet Epidemiol 20:4–16 11119293 10.1002/1098-2272(200101)20:1<4::AID-GEPI2>3.0.CO;2-T

[CR46] Selzam S, Ritchie SJ, Pingault JB et al (2019) Comparing within- and between-family polygenic score prediction. Am J Hum Genet 105(2):351–363. 10.1016/j.ajhg.2019.06.006 31303263 10.1016/j.ajhg.2019.06.006 PMC6698881

[CR47] Siva N (2008) 1000 genomes project. Nat Biotechnol 26(3):256–256. 10.1038/nbt0308-256b 18327223 10.1038/nbt0308-256b

[CR48] Smith SP, Smith OS, Mostafavi H et al (2025) A litmus test for confounding in polygenic scores. BioRxiv. 10.1101/2025.02.01.63598541573954

[CR49] Sudlow C, Gallacher J, Allen N et al (2015) UK biobank: an open access resource for identifying the causes of a wide range of complex diseases of middle and old age. PLOS Med 12(3):e1001779. 10.1371/journal.pmed.1001779 25826379 10.1371/journal.pmed.1001779 PMC4380465

[CR50] Sul JH, Martin LS, Eskin E (2018) Population structure in genetic studies: confounding factors and mixed models. PLOS Genet 14(12):e1007309. 10.1371/journal.pgen.1007309 30589851 10.1371/journal.pgen.1007309 PMC6307707

[CR51] Tan T, Jayashankar H, Guan J et al (2024) Family-gwas reveals effects of environment and mating on genetic associations, 10.1101/2024.10.01.24314703

[CR52] Torkamani A, Wineinger NE, Topol EJ (2018) The personal and clinical utility of polygenic risk scores. Nat Rev Genet 19(9):581–590. 10.1038/s41576-018-0018-x 29789686 10.1038/s41576-018-0018-x

[CR53] Veller C, Coop GM (2024) Interpreting population- and family-based genome-wide association studies in the presence of confounding. PLoS Biol 22:e3002511. 10.1371/journal.pbio.3002511 38603516 10.1371/journal.pbio.3002511 PMC11008796

[CR54] Vilhj´almsson BJ, Nordborg M (2013) The nature of confounding in genome-wide association studies. Nat Rev Genet 14(1):1–2. 10.1038/nrg3382 23165185 10.1038/nrg3382

[CR55] Visscher PM, Wray NR, Zhang Q et al (2017) 10 years of GWAS discovery: biology, function, and translation. Am J Hum Genet 101(1):5–22. 10.1016/j.ajhg.2017.06.005 28686856 10.1016/j.ajhg.2017.06.005 PMC5501872

[CR56] Wang Y, Namba S, Lopera E et al (2023) Global biobank analyses provide lessons for developing polygenic risk scores across diverse cohorts. Cell Genom 3(1):100241 36777179 10.1016/j.xgen.2022.100241 PMC9903818

[CR57] Wray NR, Yang J, Hayes BJ et al (2013) Pitfalls of predicting complex traits from SNPs. Nat Rev Genet 14(7):507–515. 10.1038/nrg3457 23774735 10.1038/nrg3457 PMC4096801

[CR58] Yang J, Lee SH, Goddard ME et al (2011a) GCTA: a tool for genome-wide complex trait analysis. Am J Hum Genet 88(1):76–82. 10.1016/j.ajhg.2010.11.011 21167468 10.1016/j.ajhg.2010.11.011 PMC3014363

[CR59] Yang J, Weedon MN, Purcell S et al (2011b) Genomic inflation factors under polygenic inheritance. Eur J Hum Genet 19:807–812. 10.1038/ejhg.2011.39 21407268 10.1038/ejhg.2011.39 PMC3137506

[CR60] Yang J, Zaitlen NA, Goddard ME et al (2014) Advantages and pitfalls in the application of mixed-model association methods. Nat Genet 46(2):100–106. 10.1038/ng.2876 24473328 10.1038/ng.2876 PMC3989144

[CR61] Yao Y, Ochoa A (2023) Limitations of principal components in quantitative genetic association models for human studies. Elife. 10.7554/eLife.79238 37140344 10.7554/eLife.79238 PMC10234632

[CR62] Young AS (2024) Genome-wide association studies have problems due to confounding: are family-based designs the answer? PLOS Biol 22:e3002568. 10.1371/journal.pbio.3002568 38607978 10.1371/journal.pbio.3002568 PMC11014432

[CR63] Young AI, Benonisdottir S, Przeworski M et al (2019) Deconstructing the sources of genotype-phenotype associations in humans. Science 365:1396–1400. 10.1126/science.aax3710 31604265 10.1126/science.aax3710 PMC6894903

[CR64] Zeng J, De Vlaming R, Wu Y et al (2018) Signatures of negative selection in the genetic architecture of human complex traits. Nat Genet 50(5):746–753. 10.1038/s41588-018-0101-4 29662166 10.1038/s41588-018-0101-4

[CR65] Zhang Y, Pan W (2015) Principal component regression and linear mixed model in association analysis of structured samples: competitors or complements? Genet Epidemiol 39(3):149–155. 10.1002/gepi.21879 25536929 10.1002/gepi.21879 PMC4366301

[CR66] Zheng G, Freidlin B, Gastwirth JL (2006) Robust genomic control for association studies. Am J Hum Genet 78:350–356. 10.1086/500054 16400614 10.1086/500054 PMC1380242

