# Supplementary material for: Within-sibling attenuation of polygenic risk score accuracy: investigating the effects of principal component analysis, LD score regression, and mixed model association in the UK Biobank
Source: Hum Genet. 2026 Jul 4;145(1):58. doi: 10.1007/s00439-026-02852-3 (PMC13332965; doi:10.1007/s00439-026-02852-3)
Supplement: Supplementary file 1 — Supplementary file1 (DOCX 1456 KB) [file 439_2026_2852_MOESM1_ESM.docx]

# Supporting information

## Supplementary note 1: Phenotype definitions

### Type 2 diabetes

Individuals assigned into the positive class were those with any of the following codes in “Diagnoses - main ICD10”, “Diagnoses - secondary ICD10”, or “Diagnoses - ICD10”: “E110”, “E111”, “E112”, “E113”, “E114”, “E115”, “E116”, “E117”, ”E118”, “E119”.

### Coronary artery disease

Individuals assigned into the positive class were those with any of the following codes in “Diagnoses - main ICD10”, “Diagnoses - secondary ICD10”, or “Diagnoses - ICD10”: “I200”, “I201”, “I208”, “I209”, “I210”, “I211”, “I212”, “I213”, “I214”, “I219”, “I220”, “I221”, “I228”, “I229”, “I235”, “I236”, “I238”, “I240”, “I250”, “I251”, “I252”, “I253”, “I254”, “I255”, “I256”, “I258”, “I259” and “I21.X”

### Breast cancer

Individuals assigned into the positive class were those with any of the those with self-reported cancer code 1002.

### Prostate cancer

Individuals assigned into the positive class were those with any of the those with self-reported cancer code 1044.

### Educational attainment

Codes in UKBB Qualifications Field (6138) were converted to years of education via the following mapping: (1,2,3,4,5,6,-7,-3) → (20,13,10,10,19,15,7,NA). From this, a constructed binary phenotype was generated consisting of those with ≤7 years of education (positive class) and those with >7 years of education (negative class). Dichotomizing educational attainment in this manner sacrifices information and inter- pretability. We employ it only to allow for confounding comparisons across binary disease outcomes. Results for this proxy should not be interpreted as estimates for a true binary educational phenotype.

## Supplementary note 2: Principal component analysis

All UK-Biobank participants were plotted using their first 16 PCs from data field 22009. Individuals were coloured according to their country or region of origin. Country of birth information were taken from UK Biobank data fields 1647 and 20115. Individuals with missing or unassigned data were excluded. A maximum of 1,000 randomly sampled individuals per country were kept for ease of visualization purposes. For the global plot, all non- European countries were collapsed into four continental bins —Africa, Asia, Americas, Oceania, while the UK and the four core European UN Statistical Division (UNSD) subregions were kept as distinct categories. For the regional plot, only people born in the UK constituent countries and the Republic of Ireland were included.

This analysis of the first 16 principal components derived from UK Biobank SNP data revealed clear evidence of both global and intra-European population structure. Broad ancestry differences were primarily captured by PCs 1, 6, and 11, whilst PCs 4, 7, and 8 reflected finer-scale structure within Europe (Figure S1).


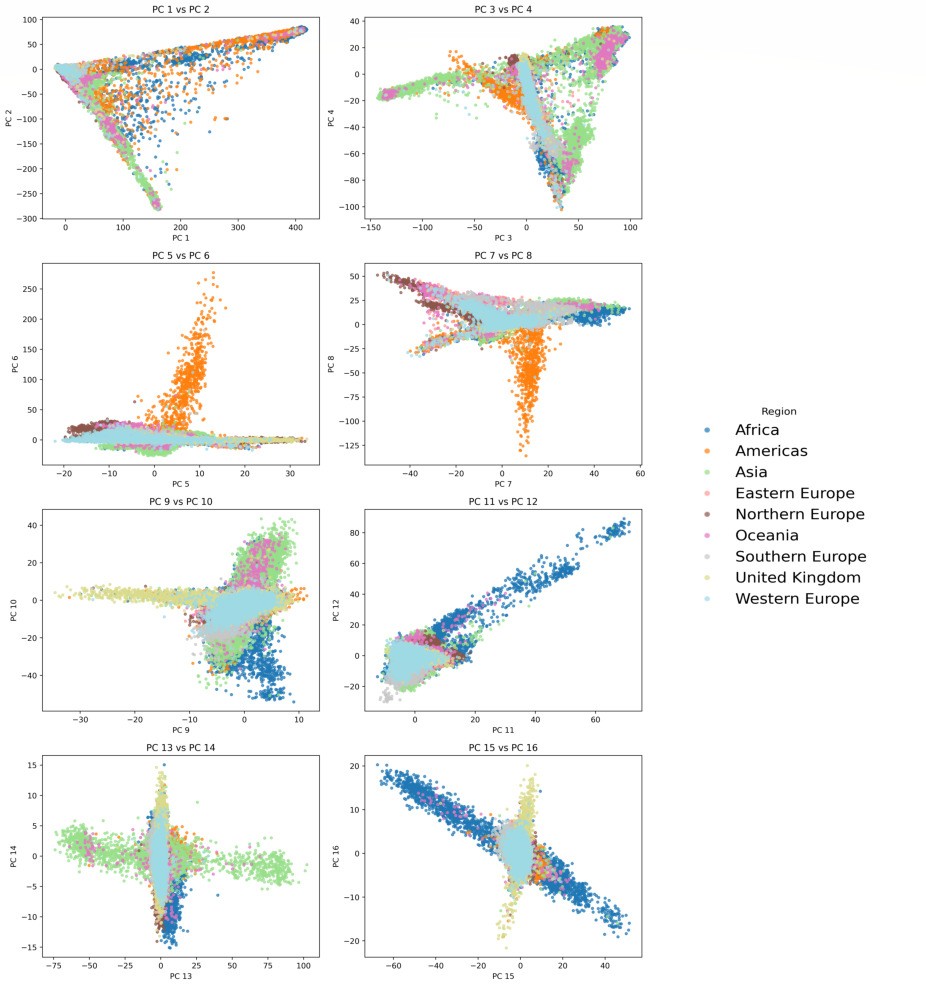


Fig. S1: PC Scores 1–16 of UK Biobank Participants

PC scores were taken from Data Field 22009 of UK Biobank. A maximum of 1,000 individuals were included per country. Individuals not assigned to any country or population were removed.

When the analysis was restricted to participants born in the UK and Ireland, addi- tional regional clustering was evident. Although the first four principal components did not appear to pull out specific sub-regions, PCs 5, 9, 11, and 14 showed distinct separation among the constituent countries of the UK and Ireland (Fig. S2).

## Association between PCs and phenotypes GWAS/PRS results with recomputed PCs

The first 16 PCs were re-calculated on the post-QC self-reported white dataset using PLINK2’s approximate PCA method (Chang et al. 2015; Galinsky et al. 2016). 126,575


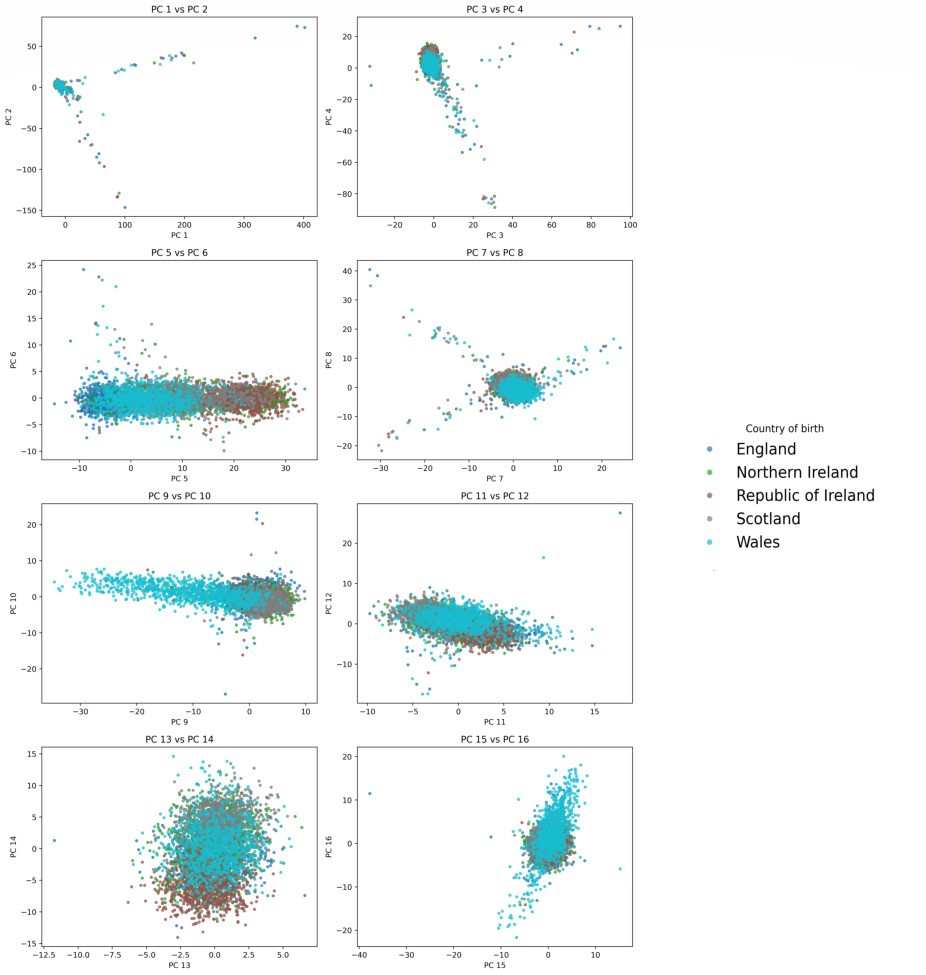


Fig. S2: PC Scores 1–16 of UK Biobank Participants (UK and Ireland Only)

PC scores were taken from Data Field 22009 of UK Biobank. A maximum of 1,000 individuals were included per country.

Table S1: Linear regression results between PCs and each phenotype. P-values are shown with significant PCs in bold.

|  | Coronary Artery Disease | Type 2 Diabetes | Prostate Cancer | Breast Cancer | Educational Attainment |
| --- | --- | --- | --- | --- | --- |
| PC1 | 7.7e-01 | 2.0e-03 | 9.6e-01 | 2.1e-01 | 9.7e-05 |
| PC2 | 9.2e-01 | 7.1e-01 | 3.2e-01 | 8.7e-01 | 5.1e-04 |
| PC3 | 1.2e-01 | 5.6e-03 | 1.5e-01 | 3.3e-01 | 8.3e-01 |
| PC4 | 1.4e-04 | 4.4e-02 | 1.6e-01 | 2.7e-01 | 8.6e-50 |
| PC5 | 2.3e-05 | 2.0e-05 | 2.7e-06 | 4.8e-01 | 6.5e-26 |
| PC6 | 1.0e-01 | 4.2e-04 | 6.9e-01 | 1.5e-01 | 3.4e-03 |
| PC7 | 2.8e-01 | 1.7e-02 | 4.4e-01 | 4.9e-01 | 3.0e-48 |
| PC8 | 2.6e-05 | 7.5e-03 | 4.9e-03 | 2.2e-02 | 2.0e-02 |
| PC9 | 4.6e-16 | 5.4e-01 | 9.7e-02 | 9.0e-01 | 3.0e-117 |
| PC10 | 5.8e-03 | 7.3e-02 | 7.5e-01 | 2.3e-01 | 3.3e-03 |
| PC11 | 2.2e-16 | 1.4e-14 | 7.0e-01 | 9.4e-01 | 1.4e-38 |
| PC12 | 1.4e-01 | 3.5e-03 | 9.7e-01 | 5.9e-02 | 7.4e-02 |
| PC13 | 8.2e-02 | 3.1e-01 | 9.9e-01 | 2.0e-01 | 5.4e-01 |
| PC14 | 8.1e-03 | 5.2e-02 | 5.5e-01 | 3.9e-02 | 1.6e-03 |
| PC15 | 9.0e-01 | 3.4e-01 | 2.3e-01 | 3.1e-01 | 3.4e-01 |
| PC16 | 1.9e-02 | 5.5e-04 | 2.1e-01 | 1.3e-01 | 2.4e-31 |

pruned SNPs were used as input for this PCA using a window-size of 50kb, step- size of 5bp and squared correlation threshold of 0.05 (Purcell et al. 2007). The HLA region was excluded during pruning. A standard GLM-based GWAS and PRS were performed as in the main text using these re-computed 16 PCs as well as age and sex as covariates.

Table S2: PRS with recomputed PCs: Classification accu- racy (%) in standard GLM-based GWAS-based PRS per- formance on discordant sibling pairs in the UK Biobank. Age, sex and the first 16 recomputed PCs were included in the GWAS and PRS.

| Phenotype | 1K SNPs | 10K SNPs | 100K SNPs |
| --- | --- | --- | --- |
| Coronary Artery Disease | 56.07 | 55.11 | 56.07 |
| Type 2 Diabetes | 59.24 | 59.75 | 58.28 |
| Breast Cancer | 53.51 | 54.68 | 54.18 |
| Prostate Cancer | 56.46 | 61.90 | 53.74 |

Table S3: PRS with recomputed PCs: Classification accu- racy (%) in standard GLM-based GWAS-based PRS perfor- mance on non-sibling discordant pairs in the UK Biobank. Age, sex and the first 16 recomputed PCs were included in the GWAS and PRS.

| Phenotype | 1K SNPs | 10K SNPs | 100K SNPs |
| --- | --- | --- | --- |
| Coronary Artery Disease | 57.45 | 58.39 | 57.29 |
| Type 2 Diabetes | 62.30 | 63.54 | 64.25 |
| Breast Cancer | 58.36 | 55.69 | 54.35 |
| Prostate Cancer | 61.22 | 56.46 | 61.22 |

## Supplementary note 3: Effects of number of included PCs on the genomic inflation factor λ and LDSC ratio

Table S4: Genomic inflation factor (λ) of GLM- and GLMM-based GWAS.

| Phenotype | GLM | GLM+16PC | GLMM+16PC |
| --- | --- | --- | --- |
| Coronary Artery Disease | 1.191 | 1.153 | 1.152 |
| Type 2 Diabetes | 1.232 | 1.214 | 1.211 |
| Breast Cancer | 1.040 | 1.037 | 1.037 |
| Prostate Cancer | 1.043 | 1.039 | 1.036 |
| Educational Attainment | 1.737 | 1.324 | 1.317 |


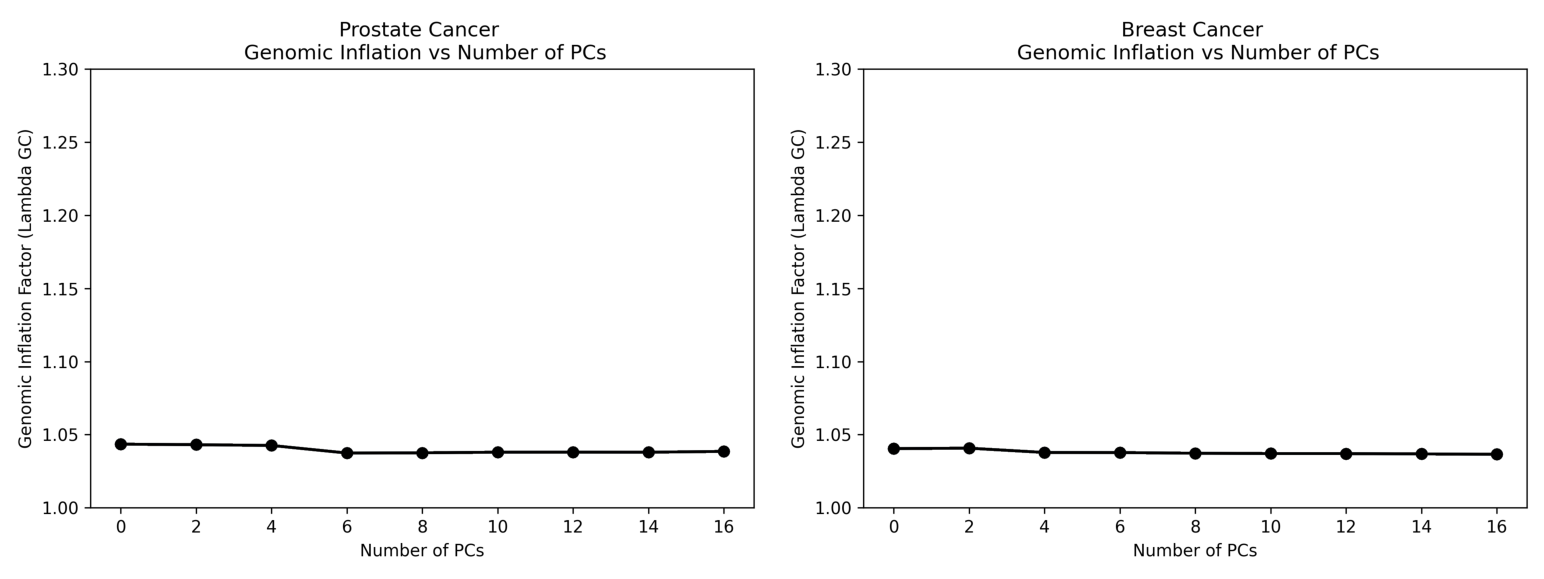


(a) Prostate Cancer (b) Breast Cancer


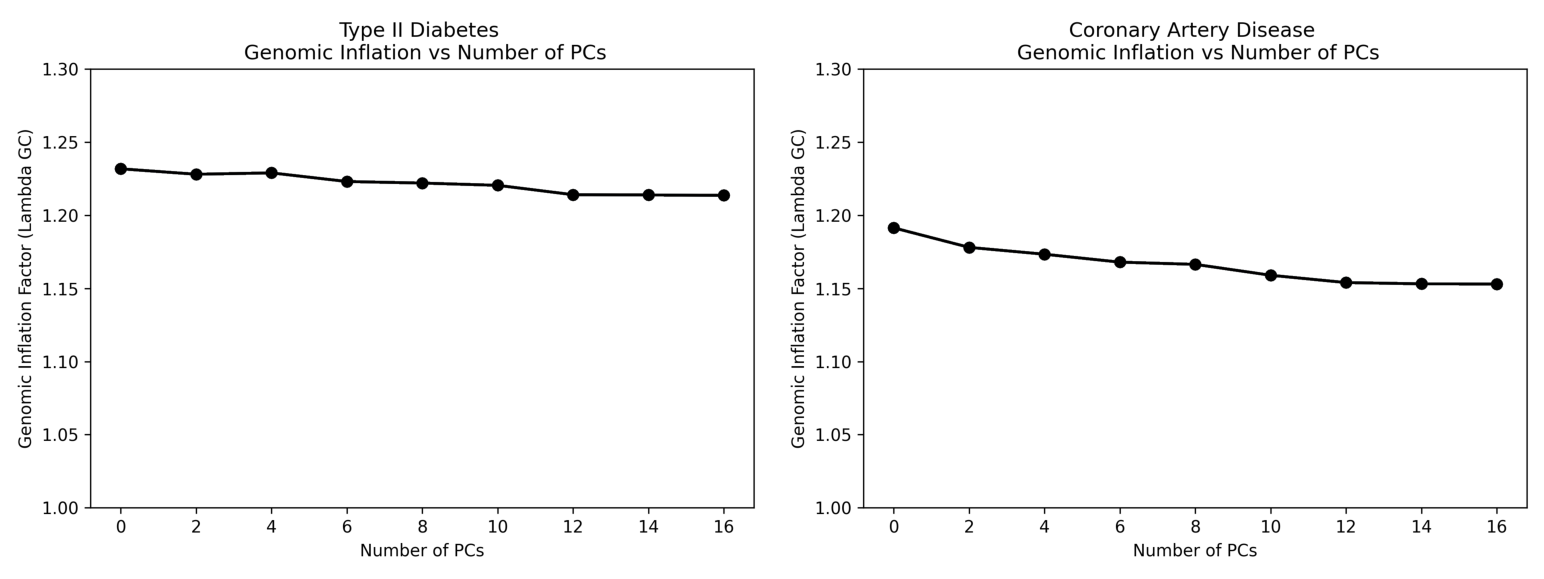


(c) Type 2 Diabetes (d) Coronary Artery Disease


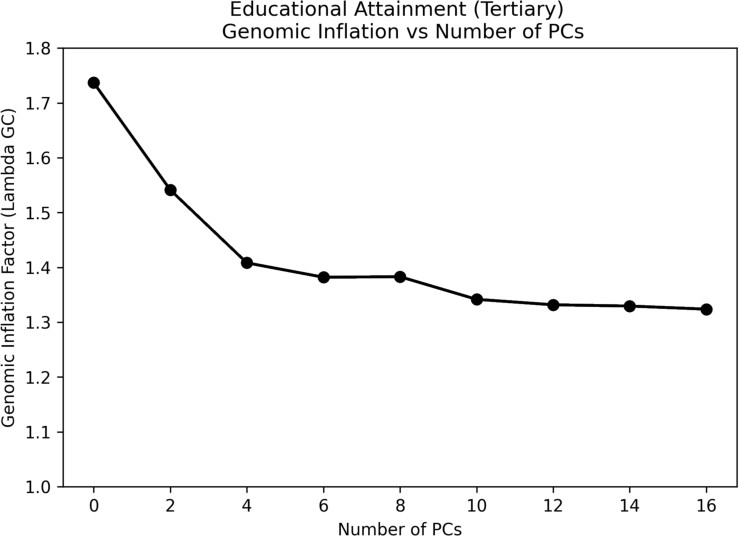


(e) Educational Attainment

Fig. S3: Effects of PCs on the genomic inflation factor λ. Summary statistics to calculate λ were generated from GLM-based GWAS. Note that the y-axis limit is higher for the educational attainment phenotype due to additional confounding.


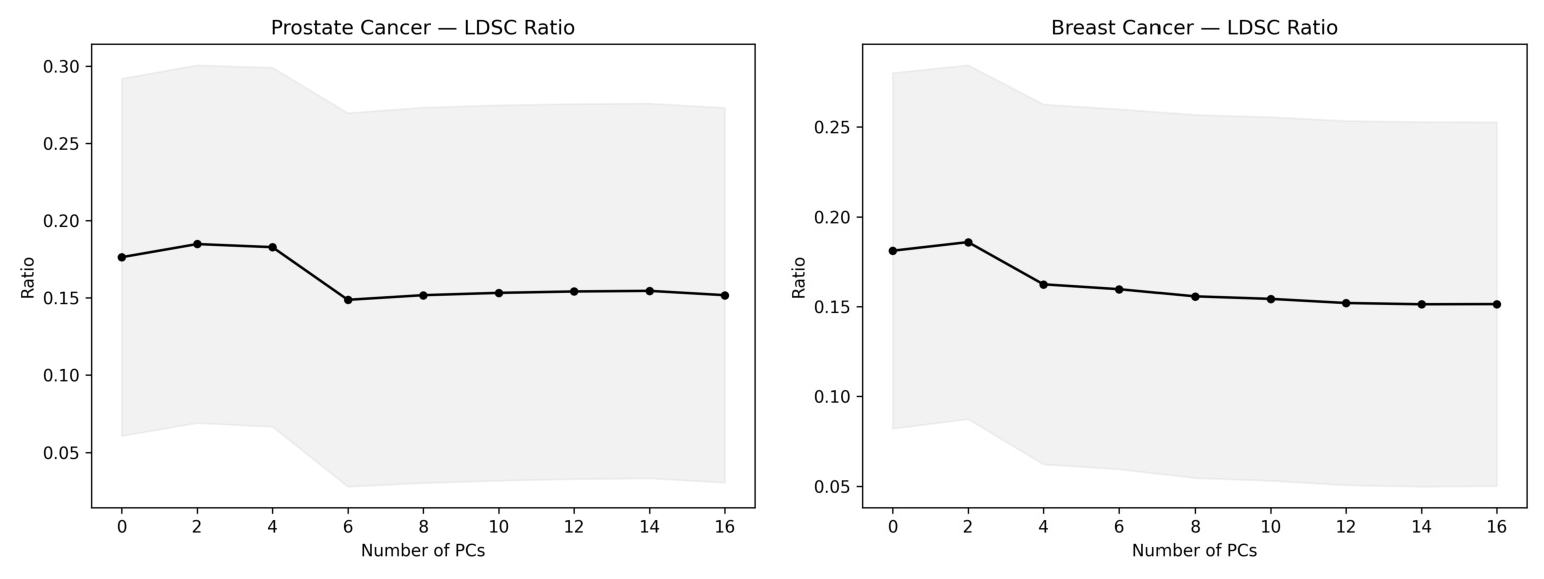


(a) Prostate Cancer (b) Breast Cancer


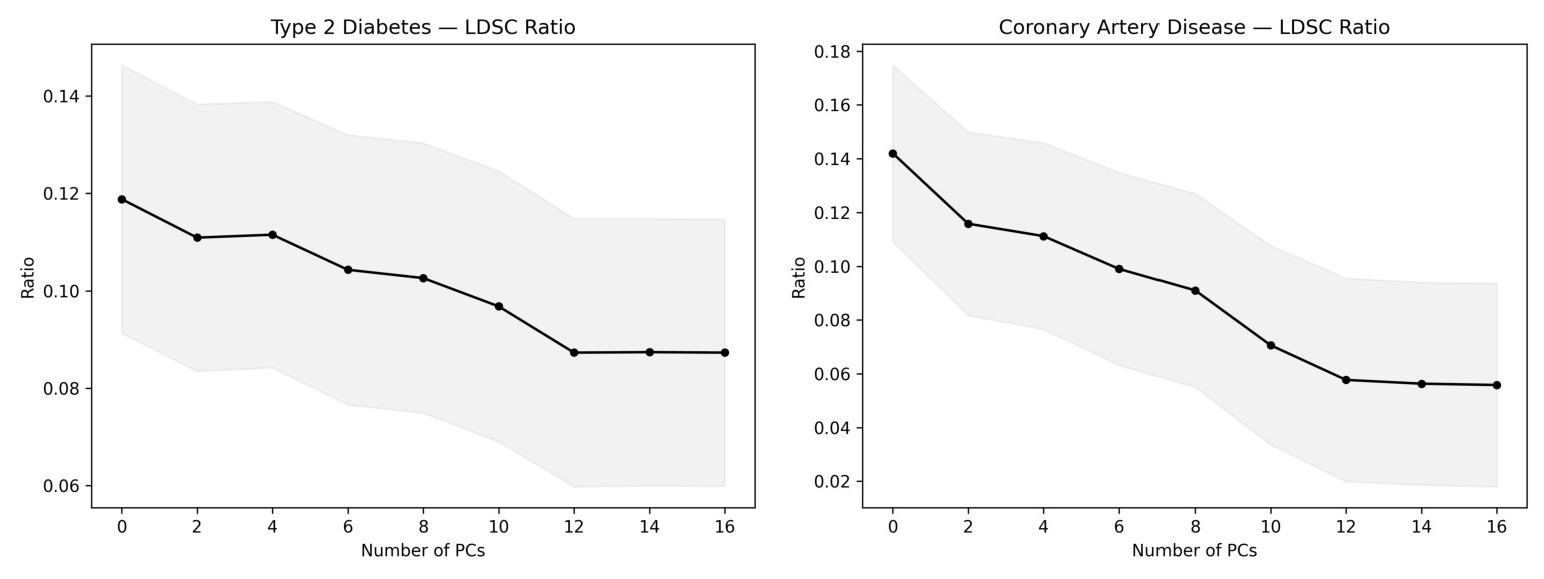


(c) Type 2 Diabetes (d) Coronary Artery Disease


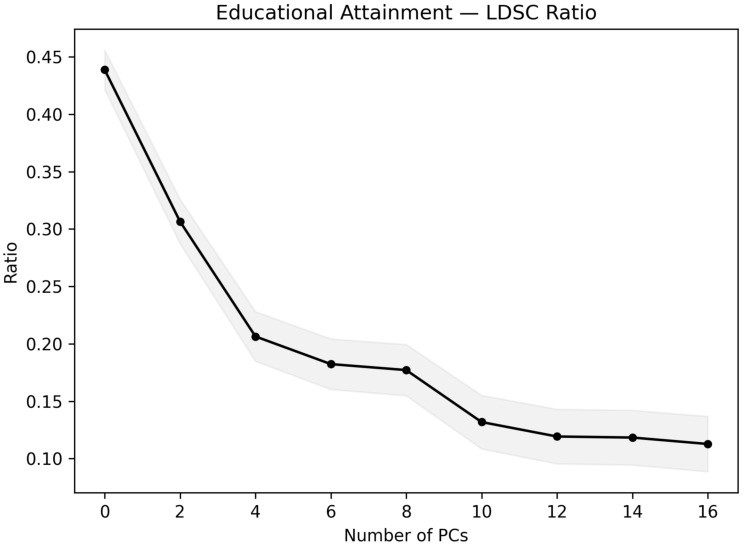


(e) Educational Attainment

Fig. S4: Effect of increasing PC inclusion on the LD-score regression (LDSR) ratio. The ratio is calculated as: (Intercept − 1)/(mean(χ^2^) − 1). Summary statistics to perform LDSC were taken from GLM-based GWAS. The standard error is indicated by shading.

##

## Coronary Artery Disease


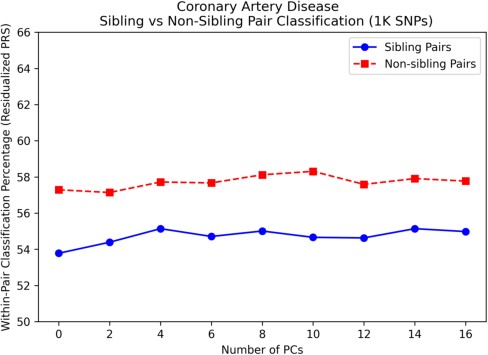

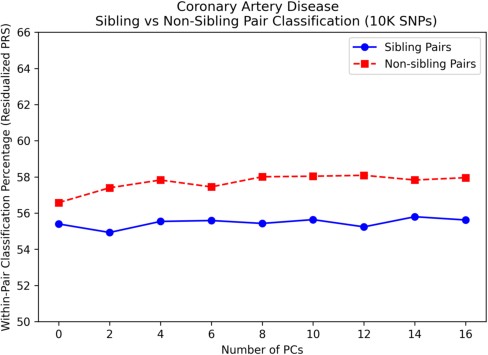

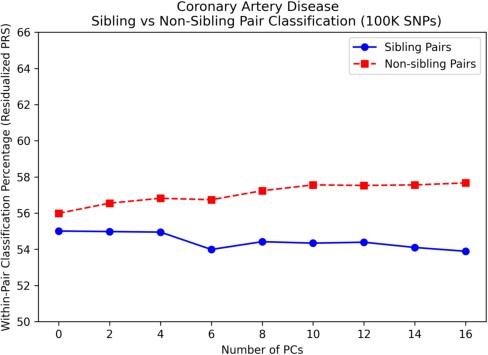


(a) 1K SNPs (b) 10K SNPs (c) 100K SNPs

Fig. S5: PRS Results for Coronary Artery Disease. PRS were derived from GLM- based GWAS. Predictive performance among sibling and non-sibling pairs with increasing inclusion of PCs is shown.

## Type 2 Diabetes


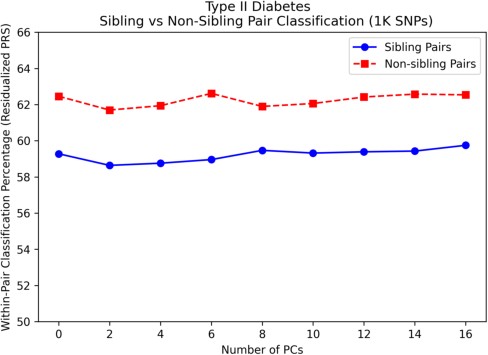

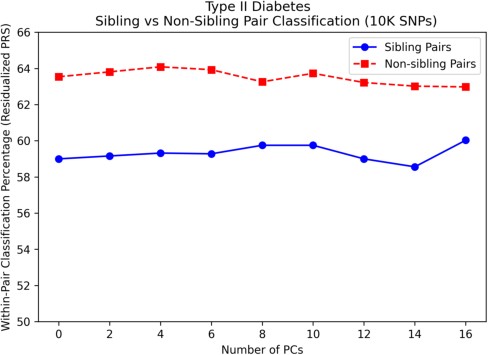

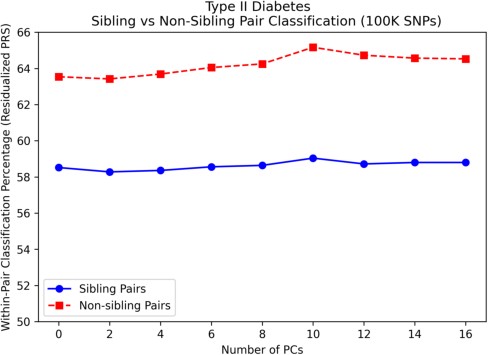


(a) 1K SNPs (b) 10K SNPs (c) 100K SNPs

Fig. S6: PRS Results for Type 2 Diabetes. PRS were derived from GLM-based GWAS. Predictive performance among sibling and non-sibling pairs with increasing inclusion of PCs is shown

Supplementary note 4: Effects of number of included PCs on predicitve performance across all SNP-set sizes

Supplementary note 5: Effects of number of included PCs on attenuation across all SNP-set sizes

Supplementary note 6: Full GLM and GLMM results

## Breast Cancer


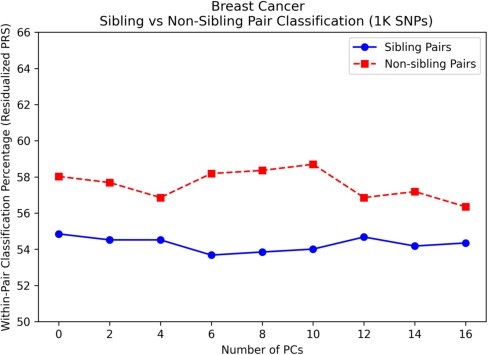

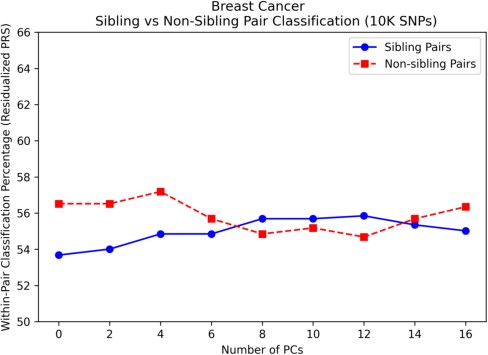

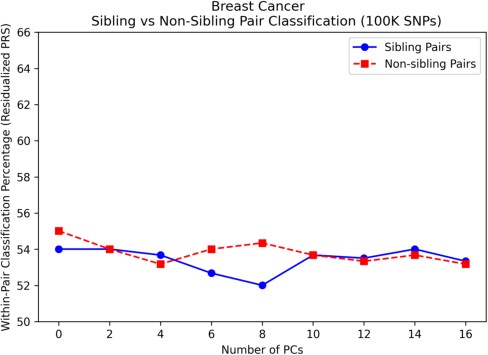


(a) 1K SNPs (b) 10K SNPs (c) 100K SNPs

Fig. S7: PRS Results for Breast Cancer. PRS were derived from GLM-based GWAS. Predictive performance among sibling and non-sibling pairs with increasing inclusion of PCs is shown

## Prostate Cancer


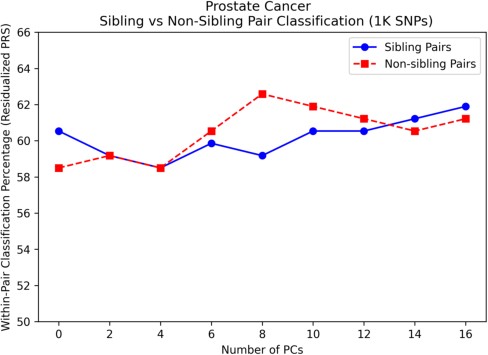

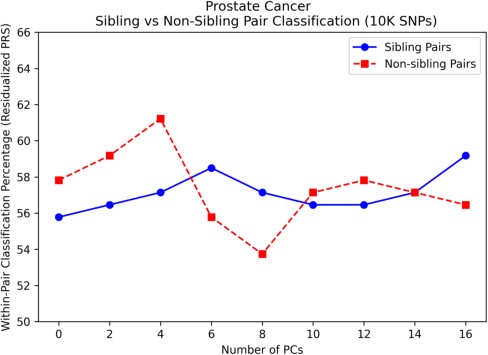

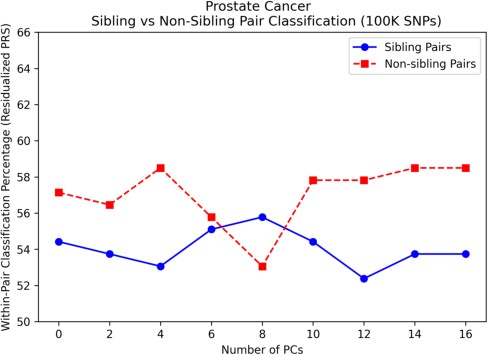


(a) 1K SNPs (b) 10K SNPs (c) 100K SNPs

Fig. S8: PRS Results for Prostate Cancer. PRS were derived from GLM-based GWAS. Predictive performance among sibling and non-sibling pairs with increasing inclusion of PCs is shown.

## Educational Attainment


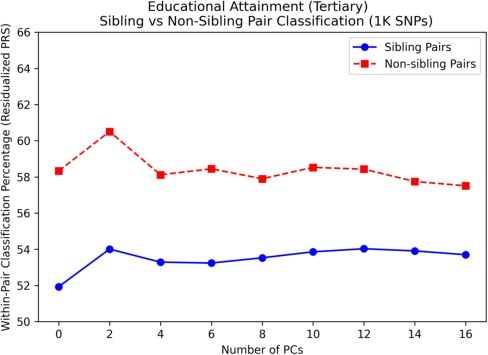

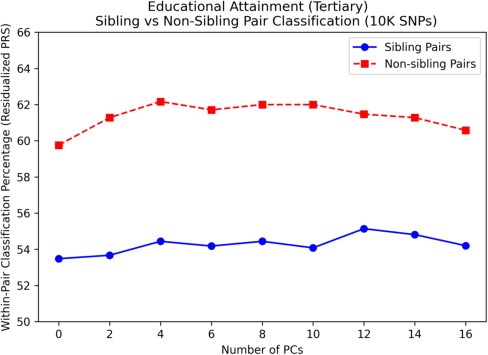

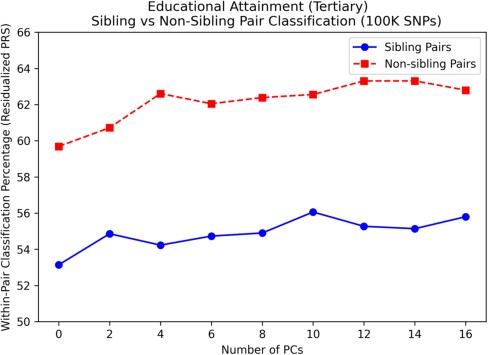


(a) 1K SNPs (b) 10K SNPs (c) 100K SNPs

Fig. S9: PRS Results for binarized Educational Attainment. PRS were derived from GLM-based GWAS. Predictive performance among sibling and non-sibling pairs with increasing inclusion of PCs is shown.

Table S5: Classification accuracy for standard GWAS-based PRS per- formance on sibling and non-sibling (population-level) discordant pairs in the UK Biobank. The full 16 PCs were included in both the GLM-based GWAS and PRS.

| Phenotype | SNP-set size Classification accuracy (%) | | | |
| --- | --- | --- | --- | --- |
|  |  | Non-sibling pairs | Sibling pairs |  |
| Coronary Artery Disease | 1K | 57.77 | 54.98 |  |
|  | 10K | 57.96 | 55.62 |  |
|  | 100K | 57.67 | 53.89 |  |
| Type 2 Diabetes | 1K | 62.54 | 59.75 |  |
|  | 10K | 62.98 | 60.03 |  |
|  | 100K | 64.53 | 58.80 |  |
| Breast Cancer | 1K | 56.35 | 54.35 |  |
|  | 10K | 56.35 | 55.02 |  |
|  | 100K | 53.18 | 53.34 |  |
| Prostate Cancer | 1K | 61.22 | 61.90 |  |
|  | 10K | 56.46 | 59.18 |  |
|  | 100K | 58.50 | 53.74 |  |
| Educational Attainment | 1K | 57.51 | 53.70 |  |
|  | 10K | 60.58 | 54.20 |  |
|  | 100K | 62.80 | 55.80 |  |


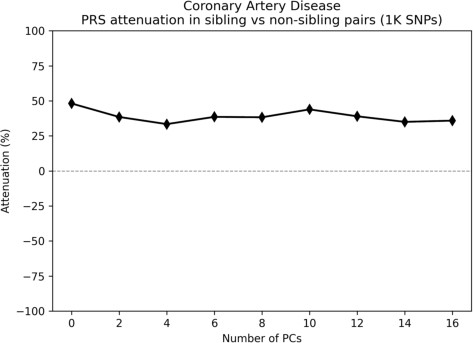

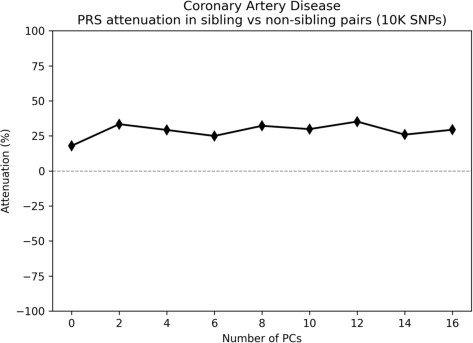

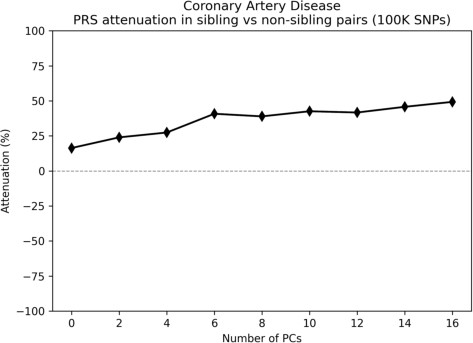


1. Coronary Artery Disease


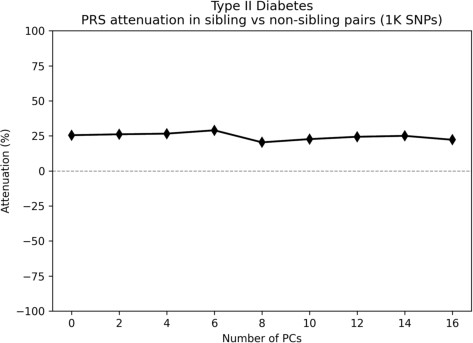

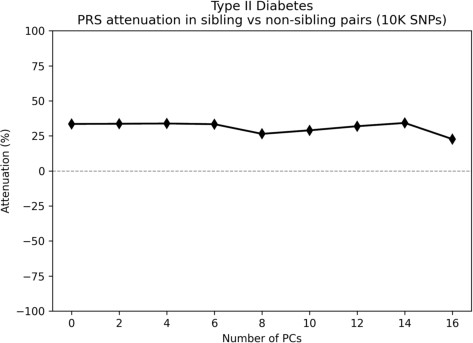

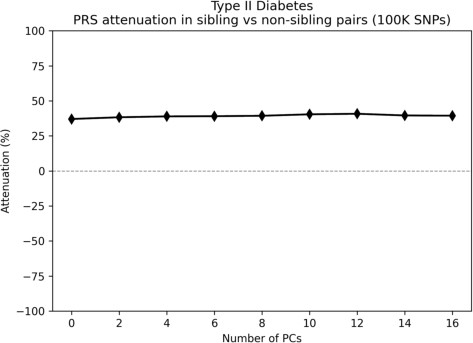


1. Type 2 Diabetes


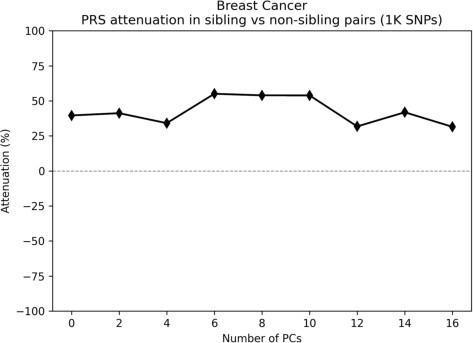

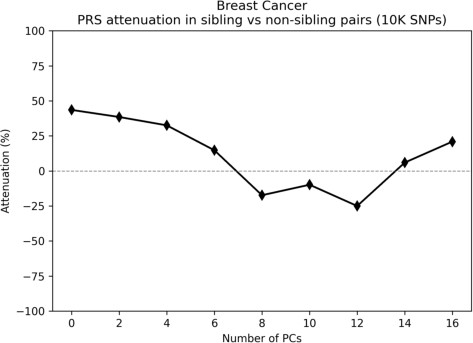

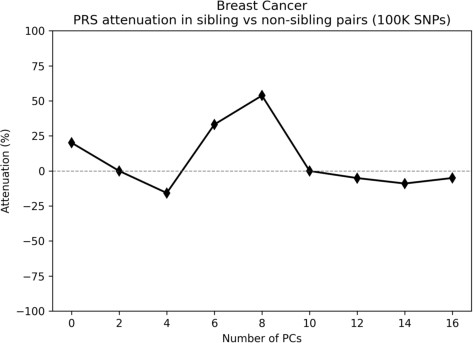


1. Breast Cancer


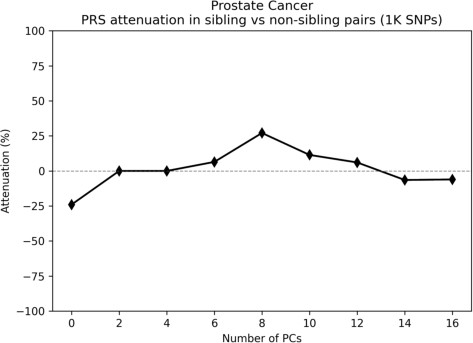

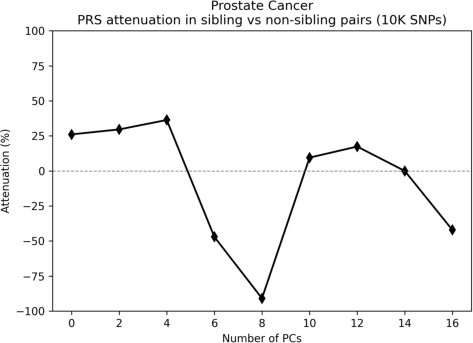

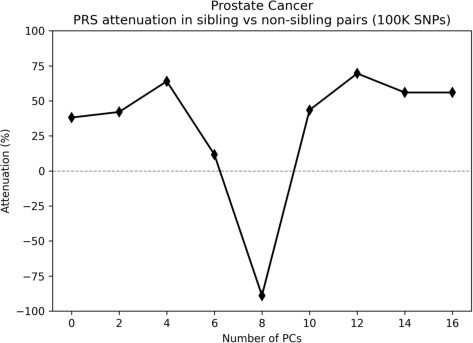


1. Prostate Cancer


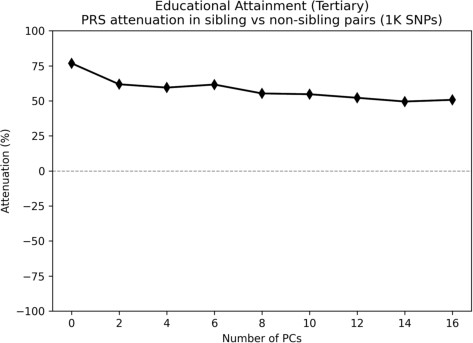

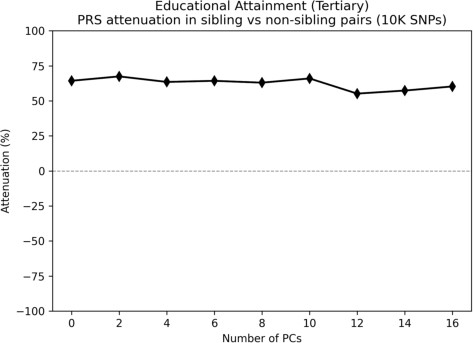

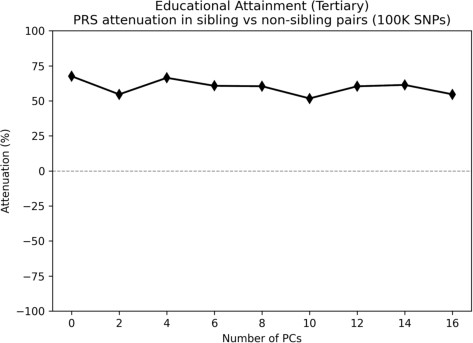


1. Educational Attainment

Fig. S10: Attenuation in predictive perfor3m5ance with increasing PC inclusion between sibling and non-sibling (population-level) pairs across phenotypes. PRS were derived from GLM-based GWAS.

Table S6: Classification accuracy for GLMM-based GWAS PRS performance on sibling and non-sibling (population-level) discordant pairs in the UK Biobank. The full 16 PCs were included in both the GLMM-GWAS and PRS.

| Phenotype | SNP-set size | Classification ac  *Non-sibling pairs* | curacy (%)  *Sibling pairs* | Attenuation (%) |
| --- | --- | --- | --- | --- |
| Coronary Artery Disease | 1K | 57.72 | 54.98 | 35.5 |
|  | 10K | 57.77 | 55.62 | 27.7 |
|  | 100K | 57.69 | 53.94 | 48.8 |
| Type 2 Diabetes | 1K | 62.70 | 59.67 | 23.9 |
|  | 10K | 63.02 | 59.83 | 24.5 |
|  | 100K | 64.49 | 59.00 | 37.9 |
| Breast Cancer | 1K | 55.69 | 54.85 | 14.8 |
|  | 10K | 55.02 | 53.68 | 26.7 |
|  | 100K | 55.85 | 54.52 | 6.8 |
| Prostate Cancer | 1K | 59.18 | 61.22 | -22.2 |
|  | 10K | 53.74 | 61.22 | -200.0 |
|  | 100K | 58.50 | 56.46 | -24.0 |
| Educational Attainment | 1K | 57.51 | 53.62 | 51.8 |
|  | 10K | 60.72 | 54.18 | 61.0 |
|  | 100K | 62.87 | 55.58 | 56.6 |
